# β-Lactamase inhibition profile of new amidine-substituted diazabicyclooctanes

**DOI:** 10.3762/bjoc.17.60

**Published:** 2021-03-12

**Authors:** Zafar Iqbal, Lijuan Zhai, Yuanyu Gao, Dong Tang, Xueqin Ma, Jinbo Ji, Jian Sun, Jingwen Ji, Yuanbai Liu, Rui Jiang, Yangxiu Mu, Lili He, Haikang Yang, Zhixiang Yang

**Affiliations:** 1 Ningxia Centre of Organic Synthesis and Engineering Technology, Ningxia Academy of Agriculture and Forestry Sciences, No. 590, Huanghe East Road, Jinfeng District, Yinchuan, Ningxia 750002, P.R. China; 2 College of Pharmacy, Ningxia Medical University, Shengli Street, Xingqing District, Yinchuan, Ningxia 750004, P.R. China

**Keywords:** amidine, antibacterial activity, β-lactamase inhibitors, diazabicyclooctane, synthesis

## Abstract

The diazabicyclooctane (DBO) scaffold is the backbone of non-β-lactam-based second generation β-lactamase inhibitors. As part of our efforts, we have synthesized a series of DBO derivatives **A1**–**23** containing amidine substituents at the C2 position of the bicyclic ring. These compounds, alone and in combination with meropenem, were tested against ten bacterial strains for their antibacterial activity in vitro. All compounds did not show antibacterial activity when tested alone (MIC >64 mg/L), however, they exhibited a moderate inhibition activity in the presence of meropenem by lowering its MIC values. The compound **A12** proved most potent among the other counterparts against all bacterial species with MIC from <0.125 mg/L to 2 mg/L, and is comparable to avibactam against both *E. coli* strains with a MIC value of <0.125 mg/L.

## Introduction

Survival stress posed by antimicrobial agents triggers multiple mechanisms [1] in microorganisms ultimately leading to the initiation of antibiotic resistance and survival of the microorganisms [2]. In case of Gram-negative pathogenic bacteria, production of β-lactamases [3] is the main arsenal of these microorganisms against antibiotics. The number of β-lactamases is increasing day by day thereby indicating the strength of these pathogens in compromising the efficacy of new antibiotics after a certain period of time. Recently, the WHO warned about the seriousness of carbapenemase-resistant Gram-negative bacteria as a global threat and urged for the development of new remedies [4].

β-Lactams (BL) have served as the first line antibiotics since the introduction of penicillin. However, due to existence and continuous increase in β-lactamases [5], multidrug therapy is becoming the new modality of bacterial treatment against multiple-drug resistant (MDR) bacteria. Multidrug therapy employs the combination of an existing antibiotic with a β-lactamase inhibitor (BLI). A few of BLI/BL combinations have been approved [6] so far for clinical applications by different countries, clavulanic acid/amoxicillin (augmentin) [7–9] being the first one, while others are in clinical trials [6]. Although augmentin [10] was successfully applied to treat the infections caused by bacterial strains producing Ambler class A and extended spectrum β-lactamases (ESBLs) [11], the emergence of new and mutant class A β-lactamases compromised its effectiveness overtime [10,12]. Subsequently, sulbactam and tazobactam [13] evolved as the BLIs of class A, B and few of class D β-lactamases [14]. These inhibitors were advantageous to clavulanic acid due to their lack of chromosomal induction of AmpC but found susceptible to a few of class A enzymes such as TEM type [10] and CTX-M (ESBL), identified in *Escherichia coli* clinical isolates [14–15].

The diazabicyclooctane (DBO) [16] ring suggested as an alternative to the β-lactam ring [17] by the Hoechst researchers [16] could not prove its antibacterial strength in early experiments rather it showed β-lactamase inhibition activity. This discovery led the researchers to develop second generation β-lactamase inhibitors, finally succeeded with the approval of avibactam and relebactam as non-β-lactam-based BLIs. Avibactam proved potent as inhibitor of *Klebsiella pneumoniae* carbapenemases (KPCs), AmpCs and some of class D β-lactamases [18] is now in clinical practice in combination with ceftazidime [6]. Followed by avibactam, a relebactam/imipenem/cilastatin [6] combination has been approved by the FDA for the treatment of clinical indications against carbapenemases, ESBLs, and MDR *Enterobacteriaceae* as well as *Pseudomonas aeruginosa* [19–21]*.* Of note, these combinations are not effective against class B metallo-lactamases and most of class D (OXA) β-lactamases. Therefore, several other DBO-based BLIs [17], such as durlobactam, nacubactam [22], zidebactam, ETX0282, ARX-1796 (a prodrug of avibactam) [23], and WCK 4234 [18,24] are passing through phase I and phase III clinical trials [6,25] in combination with different types of β-lactams.

These multidrug combinations have shown promise for future antibiotic regime and drug development based on non-β-lactam inhibitors. Nonetheless, a partial loss of activity has been reported in case of the ceftazidime/avibactam combination due to overproduction of AmpC cephalosporinases [26]. In another report it has been concluded that ESBLs of the GES, PER and BEL types in *E. coli* and *P. aeruginosa* conferred resistance against sulbactam and avibactam combinations [27]. Therefore, it is utmost necessary to continue the struggle with exploring new inhibitors capable of improved resistance and activity against all classes of β-lactamases. Based on our ongoing efforts towards the synthesis of new DBO-based BLIs, we have synthesized a number of amidine-conjugated derivatives of avibactam. Herein, we report the synthesis, and antibacterial as well as inhibitory activities of these compounds, alone and in combination with meropenem (MER), in comparison to avibactam alone and its combination with MER, an existing antibiotic in clinics.

## Results and Discussion

### Synthesis of intermediates **1–5**

**Scheme 1:**
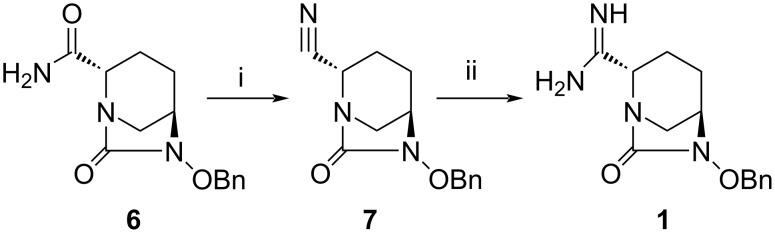
Synthesis of intermediate **1**. Reagents and conditions: (i) trifluoroacetic anhydride, CH_2_Cl_2_, 0–35 °C, 3 h, 64%; (ii) Al(Me)_3_, NH_4_Cl, CH_2_Cl_2_, 0 °–rt, 16 h, 44%.

The preparation of intermediate **1** is the key step for the synthesis of the final compounds (Scheme 1). Compound **1** was synthesized by dehydration of amide [28] **6** which is commercially available. Dehydration was achieved by reacting **6** with trifluoroacetic anhydride in CH_2_Cl_2_ at room temperature (rt) and is described elsewhere [18]. Conversion of the cyano compound **7** into the corresponding amidine compound **1**, the key intermediate, proved cumbersome. Several experiments and reagents [29] were tried before finding trimethylaluminum (Al(Me)_3_) and NH_4_Cl [30] as the reagents of choice for this conversion. As a result, compound **7** was reacted with Al(Me)_3_ and NH_4_Cl to furnish amidine **1** in CH_2_Cl_2_ starting the reaction at low temperature followed by increasing the temperature to ambient temperature for 16 h. Amidine **1** was obtained in 44% yield after purification by column chromatography using MeOH and CH_2_Cl_2_. The low yield of this reaction was due to the formation of two isomeric products as revealed by TLC and subsequent analysis by analytical LC–MS. The NMR spectra of both isomers, after chromatographic separation, showed different chemical shifts for the protons at C2 position of the DBO ring, indicating a racemization during the reaction process. The less polar isomer with *R*-configuration [31–32] at C2 showed a complete loss of β-lactamase inhibition activity as compared to the more polar isomer. Therefore, the less polar isomer was discarded while saving the more polar *S*-isomer, (relative ratio of *S*:*R* isomers = 6:1).

**Scheme 2:**
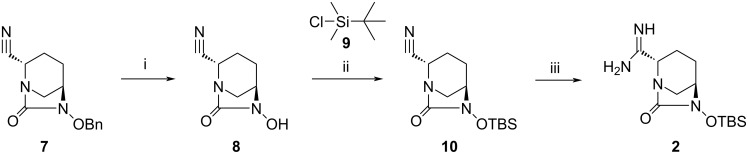
Synthesis of intermediate **2**. Reagents and conditions: (i) Pd/C (wet), EtOAc/CH_2_Cl_2_, H_2_, 45 psi, rt, 2 h, 95%; (ii) imidazole, CH_2_Cl_2_, rt, 16 h, 46%; (iii) Al(Me)_3_, NH_4_Cl, CH_2_Cl_2_, 0 °C-rt, 40 h, 23%.

The synthesis of intermediate **2** started from the hydrogenation of **7** by following a previously described method using *N,N*-dimethylformamide (DMF)/CH_2_Cl_2_ [23] as solvent led to a low yield in our hands. Therefore, we planned to switch the solvent from DMF to EtOAc whereupon the yield improved, however, still an amino derivative as side product was observed. The addition of CH_2_Cl_2_ with ethyl acetate proved helpful in increasing the yield and the NMR of the crude product **8** was acceptable to use it for further reaction without purification. The hydroxy group in **8** was then protected by TBS (*tert*-butyldimethylsilane) using *tert*-butyldimethylsilyl chloride (**9**, TBSCl) and imidazole in CH_2_Cl_2_. The thus obtained derivative **10** was then subjected to an amidination by Al(Me)_3_ and NH_4_Cl to afford amidine **2** (Scheme 2).

**Scheme 3:**
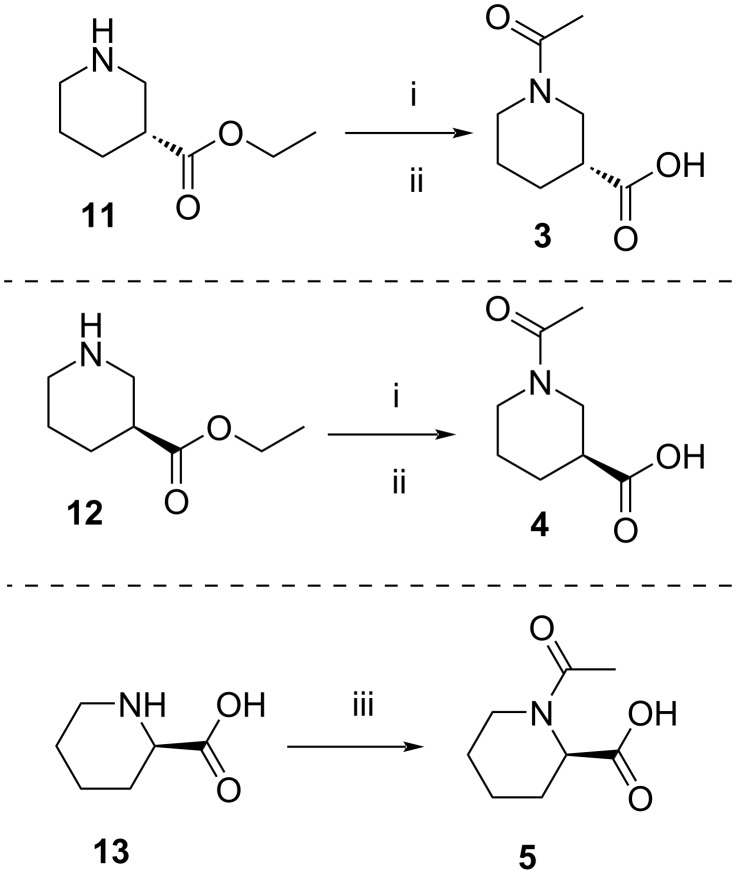
Synthesis of intermediates **3**–**5**. Reagents and conditions: (i) (Ac)_2_O, CH_2_Cl_2_, rt, 24 h, 95–99%; (ii) aqueous NaOH, 0 °C, 2 h, 85–99%; (iii) (Ac)_2_O, H_2_O, rt, 3 h, 68%.

Compounds **3** and **4** were prepared from commercially available compounds **11** and **12**, respectively, in two steps. In the first step, the ester derivatives were N-acylated by acetic anhydride in CH_2_Cl_2_ followed by hydrolysis using aqueous NaOH in THF to afford the required intermediates **3** and **4** in overall good yields. Compound **5** was obtained by direct acylation of the commercially available acid **13** using acetic anhydride and a stoichiometric amount of water at room temeprature (Scheme 3).

### Synthesis of compounds **A1–23**

**Scheme 4:**
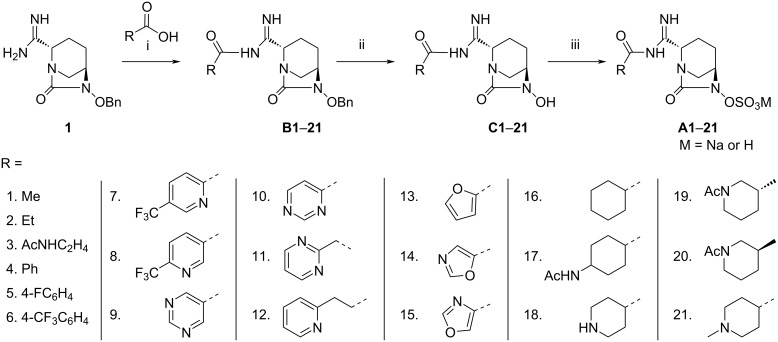
Synthesis of compounds **A1–21**. Reagents and conditions: (i) acetyl chloride, TEA, CH_2_Cl_2_, rt, 16 h, 97% (for **B1**), HATU, DIPEA or DCC, DMAP, DMF or THF, rt, 16–24 h, 43–98%. (ii) Pd/C (wet), THF or EtOAc/TEA, H_2_, rt, 16 h, 22–96%; (iii) SO_3_·pyridine, pyridine, or SO_3_·pyridine, TEA, THF/water, rt, 16 h, or then Dowex-50wx Na^+^, 8–99%.

The synthesis of compounds **A1–21** starting from intermediate **1** was accomplished as depicted in Scheme 4. The coupling of the organic acids with amidine **1** to form the corresponding derivatives **B1–21** was achieved by common coupling reagents such as *N,N'*-dicyclohexylcarbodiimide (DCC) or (*O*-(7-aza-1*H*-benzotriazol-1-yl)-*N*,*N*,*N*',*N*'-tetramethyluronium hexafluorophosphate) (HATU) [33] in DMF or CH_2_Cl_2_ with either *N,N*-diisopropylethylamine (DIPEA) or 4-dimethylaminopyridine (DMAP) as the base. Then, the palladium-catalyzed hydrogenation of compounds **B1–21** in THF or EtOAc led to the hydroxy derivatives **C1–21**. It has been observed that a catalytic amount of triethylamine (TEA) in EtOAc enhances the rate of the hydrogenolysis of benzyl ethers. Compounds **C1–21** were then reacted with SO_3_·pyridine to form sulfonic acid derivatives **A1–21** after purification by preparative HPLC. The sodium salts of these compounds were obtained by ion exchange using a column filled with Dowex-50wx Na^+^ resin using water as the eluent, followed by lyophilization. In case of compound **A18**, Boc deprotection was achieved by using trifluoroacetic acid (TFA) before the preparative HPLC.

**Scheme 5:**
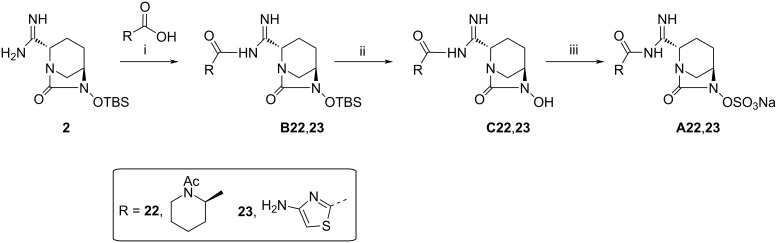
Synthesis of compounds **A22** and **A23**. Reagents and conditions: (i) HATU, DIPEA or DCC, DMAP, DMF or THF, rt, 16 h, 62% (**B22**), 80% (**B23**); (ii) TBAF, THF, 80% (**C22**), 92% (**C23**); (iii) SO_3_·pyridine, pyridine, or SO_3_·pyridine, TEA, THF/water, rt, 16 h, then Dowex-50wx Na^+^, 83% (**A22**), 35% (**A23**).

The synthesis of compounds **A22** and **A23** was accomplished by an alternative route elaborated in Scheme 5. Coupling of compound **5** and intermediate **2** was achieved by using HATU and DIPEA in a DMF/CH_2_Cl_2_ mixture to form the derivative **B22** which was treated with tetrabutylammonium fluoride (TBAF) in THF to obtain the hydroxy derivative **C22**. Compound **C22** was converted to the sodium salt of **A22** by using the procedure described for derivative **A1**. Analogously, compound **A23** was prepared starting from 4-aminothiazole-2-carboxylic acid and amidine derivative **2** (Scheme 5) according to the procedures described for **A22**.

### In vitro antibacterial efficacy

We synthesized a series of amidine derivatives of avibactam containing a variety of substituents, forming amide linkage with NH_2_ of amidine of the parent intermediate **1** or **2**. The different kinds of substituents (R) introduced in the final compounds **A1–23** are depicted in Table 1. In vitro antibacterial activities of compounds **A1–23** were determined first without combining the compounds with an antibacterial drug and the minimum inhibitor concentration (MIC) of each compound was determined for all ten bacterial strains, i.e., *E. coli* clinical isolate, *E. coli* 8739, *K. pneumoniae* clinical isolate, *K. pneumoniae* 700603, *E. cloacae* clinical isolate, *E. cloacae* 700323, *A. baumannii* clinical isolate, *A. baumannii* 19606, *P. aeruginosa* clinical isolate, and *P. aeruginosa* 9027 (Table 1). All newly synthesized compounds showed MIC values >64 mg/L against all tested bacterial species. For comparison, the MIC values of avibactam against the bacterial strains were also determined and were found comparable to the synthesized compounds (MIC >64 mg/L). This indicates that both, avibactam and compounds **A1**–**23** are not antibacterial in action when used alone. Next, we determined the antibacterial activity of meropenem (MER) alone and in combination with avibactam as well as in combination with the synthesized compounds **A1**–**23**. From Table 1, it can be deduced that the antibacterial activity of MER increased in the presence of avibactam (4 mg/L) in all bacterial strains under observation. The MIC values of MER without avibactam were observed to be in the range of 2 mg/L to 4 mg/L, whereas after the addition of avibactam the antibacterial activity changed to <0.125 mg/L–1 mg/L, indicating the enzyme inhibition effect of avibactam.

**Table 1:** In vitro antibacterial activity of avibactam and compounds **A1-23** alone as well as in combination with meropenem (MER).

		minimum inhibitory concentration (MIC, mg/L)									

		*E. coli*^a^	*E. coli*^b^	*K. p*^c^	*K. p*^d^	*E.c*^e^	*E.c*^f^	*A.b*^g^	*A.b*^h^	*P.a*^i^	*P.a*^j^

	**A1**–**23**, avibactam alone	>64	>64	>64	>64	>64	>64	>64	>64	>64	>64
	MER alone	4	4	4	2	4	4	4	2	4	4
	MER + avibactam	<0.125	<0.125	<0.125	<0.125	<0.125	<0.125	1	0.5	0.5	0.25
R:											
Me	MER + **A1**	0.5	<0.125	2	0.5	2	1	2	0.5	0.5	1
Et	MER + **A2**	0.5	<0.125	2	1	2	2	2	0.5	0.5	0.5
AcNHC_2_H_4_	MER + **A3**	<0.125	0.25	1	0.25	1	1	2	0.5	0.5	1
Ph	MER + **A4**	2	0.25	1	0.25	2	0.25	2	0.5	2	1
4-FC_6_H_4_	MER + **A5**	1	0.25	2	0.25	2	1	2	1	1	1
4-CF_3_C_6_H_4_	MER + **A6**	0.5	0.25	2	0.5	2	2	2	0.5	1	0.5
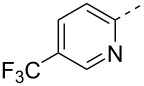	MER + **A7**	1	0.25	2	0.5	2	1	2	0.5	0.5	1
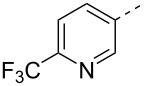	MER + **A8**	0.5	<0.125	2	0.5	2	2	2	0.5	0.5	1
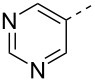	MER + **A9**	0.5	0.5	2	1	2	2	2	1	1	2
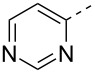	MER + **A10**	1	0.5	2	0.5	2	1	2	0.5	0.5	1
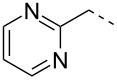	MER + **A11**	0.25	0.25	0.25	0.25	0.5	1	2	0.5	0.25	1
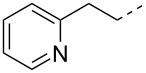	MER + **A12**	<0.125	<0.125	<0.125	<0.125	0.5	0.5	2	1	0.25	0.5
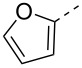	MER + **A13**	0.5	<0.125	2	0.25	2	1	2	0.5	0.5	1
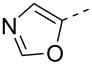	MER + **A14**	0.25	0.25	0.25	0.25	2	0.25	2	0.5	0.25	1
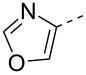	MER + **A15**	0.25	0.25	0.25	0.25	2	1	2	1	0.25	1
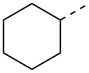	MER + **A16**	1	<0.125	2	0.25	2	0.5	2	0.5	0.5	0.5
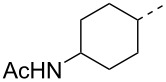	MER + **A17**	0.25	0.25	0.25	0.25	1	1	2	0.5	0.25	0.5
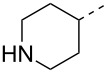	MER + **A18**	0.25	0.5	0.25	0.25	2	1	2	2	1	0.25
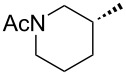	MER + **A19**	0.25	0.25	2	0.25	1	0.5	2	1	1	0.5
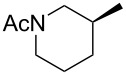	MER + **A20**	0.25	0.25	1	0.25	2	0.5	2	0.5	1	0.5
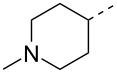	MER + **A21**	0.5	0.5	2	0.5	2	0.5	2	0.5	0.25	1
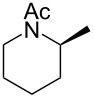	MER + **A22**	1	0.5	2	0.25	1	0.5	2	0.5	0.25	0.5
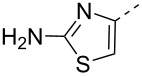	MER + **A23**	0.25	0.25	1	0.25	0.5	1	2	0.5	0.25	0.5

^a^*E. coli* clinical isolate; ^b^*E. coli* 8739; ^c^*K. pneumoniae* clinical isolate; ^d^*K. pneumoniae* 700603; ^e^*E. cloacae* clinical isolate; ^f^*E. cloacae* 700323; ^g^*A. baumannii* clinical isolate; ^h^*A. baumannii* 19606; ^i^*P. aeruginosa* clinical isolate; ^j^*P. aeruginosa* 902.

In order to establish the lactamase inhibition effect of the synthesized avibactam derivatives **A1**–**23**, we determined the antibacterial activity of MER in combination with compounds **A1**–**23** individually. The results are summarized in Table 1 as MIC values of each compound against each bactrial strain. From Table 1 it is evident that all compounds enhanced the antibacterial activity of MER (MIC <0.125 mg/L to 2 mg/L) as compared to meropenem alone (MIC 2 mg/L to 4 mg/L). Compound **A12** proved to be the most potent compound among the other counterparts against all bacterial species with MIC values from <0.125 mg/L to 2 mg/L, and is comparable to avibactam against both *E. coli* strains and *K. pneumoniae* strains with a MIC value of <0.125 mg/L. From the data in Table 1 it is evident that the *A. baumannii* clinical isolate is the most resistant strain against all newly synthesized compounds as well as against avibactam showing MIC values of 2 mg/L and 1 mg/L, respectively. However, *E. coli* 8739 was the most susceptible strain to most of the synthesized compounds for example, **A1**, **A2**, **A8**, **A12**, **A13** and **A16** with a MIC value of <0.125 mg/L.

Although a comprehensive conclusion cannot be drawn about the structure–activity relationship of the compounds, a few points could be ascertained. For example, varying the substituents (R) at the amidine attached to the C2 position of the DBO ring definitely tuned the antibacterial activity of the compounds **A1**–**23**. Further, aromatic substituents proved slightly better than alkyl substituents against most of the bacterial strains, however, stereochemically isomeric substituents (e.g., in **A19** and **A20**) did not pose a substantial effect on the activity.

## Conclusion

We have successfully synthesized a series of avibactam derivatives with substituted amidines at the C2 position of DBO in moderate to good overall yields. In vitro antibacterial testing of the compounds was performed against ten bacterial strains containing different β-lactamase enzymes. The compounds were tested alone and in combination with the existing antibiotic, meropenem. All compounds showed no antibacterial efficacy when tested alone, however, all compounds exhibited a moderate antibacterial activity in combination with meropenem. This confers the β-lactamase inhibition activity of these compounds as illustrated by reduced MIC values of meropenem in the presence of the test compounds. Compound **A12** was the most potent inhibitor in case of all bacterial strains under observation and may be a lead compound for further development.

## Supporting Information

File 1 Detailed experimental protocols, ^1^H NMR, LC–MS data, and copies of ^1^H NMR spectra of the final compounds.

## References

[1] Wencewicz T A (2019). J Mol Biol.

[2] Peterson E, Kaur P (2018). Front Microbiol.

[3] Tooke C L, Hinchliffe P, Bragginton E C, Colenso C K, Hirvonen V H A, Takebayashi Y, Spencer J (2019). J Mol Biol.

[4] Bloom D E, Cadarette D (2019). Front Immunol.

[5] Bush K, Bradford P A (2020). Clin Microbiol Rev.

[6] Butler M S, Paterson D L (2020). J Antibiot.

[7] Saudagar P S, Survase S A, Singhal R S (2008). Biotechnol Adv.

[8] Finlay J, Miller L, Poupard J A (2003). J Antimicrob Chemother.

[9] (1996). Drug Ther Bull.

[10] Blazquez J, Baquero M R, Canton R, Alos I, Baquero F (1993). Antimicrob Agents Chemother.

[11] Ghafourian S, Sadeghifard N, Soheili S, Sekawi Z (2015). Curr Issues Mol Biol.

[12] Papp-Wallace K M, Bonomo R A (2016). Infect Dis Clin North Am.

[13] Shlaes D M (2013). Ann N Y Acad Sci.

[14] Bush K, Bradford P A (2016). Cold Spring Harbor Perspect Med.

[15] Shen Z, Ding B, Bi Y, Wu S, Xu S, Xu X, Guo Q, Wang M (2017). Antimicrob Agents Chemother.

[16] Coleman K (2011). Curr Opin Microbiol.

[17] González-Bello C, Rodríguez D, Pernas M, Rodríguez Á, Colchón E (2020). J Med Chem.

[18] Papp-Wallace K M, Nguyen N Q, Jacobs M R, Bethel C R, Barnes M D, Kumar V, Bajaksouzian S, Rudin S D, Rather P N, Bhavsar S (2018). J Med Chem.

[19] Nichols W W, Newell P, Critchley I A, Riccobene T, Das S (2018). Antimicrob Agents Chemother.

[20] van Duin D, Bonomo R A (2016). Clin Infect Dis.

[21] Rodriguez B A, Girotto J E, Nicolau D P (2018). Curr Pediatr Rev.

[22] Morinaka A, Tsutsumi Y, Yamada M, Suzuki K, Watanabe T, Abe T, Furuuchi T, Inamura S, Sakamaki Y, Mitsuhashi N (2015). J Antimicrob Chemother.

[23] Gordon E M, Duncton M A J, Gallop M A (2018). J Med Chem.

[24] Mushtaq S, Vickers A, Woodford N, Livermore D M (2017). J Antimicrob Chemother.

[25] Tehrani K H M E, Martin N I (2018). Med Chem Commun.

[26] Chalhoub H, Sáenz Y, Nichols W W, Tulkens P M, Van Bambeke F (2018). Int J Antimicrob Agents.

[27] Ortiz de la Rosa J-M, Nordmann P, Poirel L (2019). J Antimicrob Chemother.

[28] Ball M, Boyd A, Ensor G J, Evans M, Golden M, Linke S R, Milne D, Murphy R, Telford A, Kalyan Y (2016). Org Process Res Dev.

[R29] 29For example BuLi/HMDS, MeOH/HCl, Trimethylsilylsulfonate were used in different experiments under various conditions.

[30] Moss R A, Ma W, Merrer D C, Xue S (1995). Tetrahedron Lett.

[R31] 31The *R*-configuration to the less polar isomer was assigend on the basis of the comparison of the NMR data with the previous literature [32] and with the antibacterial activity of this isomer [32].

[32] Abe T, Okue M, Sakamaki Y (2012). Optically active diazabicyclooctane derivatives and process for preparing the same. US Pat. Appl..

[33] Dierks A, Tönjes J, Schmidtmann M, Christoffers J (2019). Chem – Eur J.

