# Supplementary material for: β-Lactamase inhibition profile of new amidine-substituted diazabicyclooctanes
Source: Beilstein J Org Chem. 2021 Mar 12;17:711–8. doi: 10.3762/bjoc.17.60 (PMC7961884; doi:10.3762/bjoc.17.60)
Supplement: File 1 — Detailed experimental protocols, 1H NMR, LC–MS data, and copies of 1H NMR spectra of the final compounds. [file Beilstein_J_Org_Chem-17-711-s001.pdf]

BEILSTEIN JOURNAL OF ORGANIC CHEMISTRY

Supporting Information

for

β-Lactamase inhibition profile of new amidine-substituted diazabicyclooctanes

Zafar Iqbal, Lijuan Zhai, Yuanyu Gao, Dong Tang, Xueqin Ma, Jinbo Ji, Jian Sun, Jingwen Ji, Yuanbai Liu, Rui Jiang, Yangxiu Mu, Lili He, Haikang Yang and Zhixiang Yang

Beilstein J. Org. Chem. **2021,** *17,* 711–718. doi:10.3762/bjoc.17.60

Detailed experimental protocols, ^{1}H NMR, LC–MS data, and copies of ^{1}H NMR spectra of the final compounds

License and Terms: This is a supporting information file under the terms of the Creative Commons Attribution License (https://creativecommons.org/licenses/by/4.0). Please note that the reuse, redistribution and reproduction in particular requires that the author(s) and source are credited and that individual graphics may be subject to special legal provisions.
The license is subject to the *Beilstein Journal of Organic Chemistry* terms and conditions: (https://www.beilstein-journals.org/bjoc/terms)

Table of contents:

1. General methods and materials S4

2. Experimental procedures S4

2.1 (2*S*,5*R*)-6-(Benzyloxy)-7-oxo-1,6-diazabicyclo[3.2.1]octane-2-carbonitrile (**7**) S4

2.2 (2*S*,5*R*)-6-(Benzyloxy)-7-oxo-1,6-diazabicyclo[3.2.1]octane-2-carboximidamide (**1**) S4

2.3 (2*S*,5*R*)-6-Hydroxy-7-oxo-1,6-diazabicyclo[3.2.1]octane-2-carbonitrile (**8**) S4

2.4 (2*S*,5*R*)-6-((*tert*-Butyldimethylsilyl)oxy)-7-oxo-1,6-diazabicyclo[3.2.1]octane-2-carbonitrile (**10**) S5

2.5 (2*S*,5*R*)-6-((*tert*-Butyldimethylsilyl)oxy)-7-oxo-1,6-diazabicyclo[3.2.1]octane-2-carboximidamide (**2**) S5

2.6 (*R*)-1-Acetylpiperidine-3-carboxylate (**3**) S5

2.7 (*S*)-1-Acetylpiperidine-3-carboxylate (**4**) S6

2.8 (*S*)-1-Acetylpiperidine-2-carboxylic acid (**5**) S6

2.9 *N*-(((2*S*,5*R*)-6-(Benzyloxy)-7-oxo-1,6-diazabicyclo[3.2.1]octan-2-yl)(imino)methyl)acetamide (**B1**) S6

2.10 *N*-(((2*S*,5*R*)-6-Hydroxy-7-oxo-1,6-diazabicyclo[3.2.1]octan-2-yl)(imino)methyl)acetamide (**C1**) S6

2.11 (2*S*,5*R*)-2-(*N*-Acetylcarbamimidoyl)-7-oxo-1,6-diazabicyclo[3.2.1]octan-6-yl hydrogen sulfate (**A1**) S6

2.12 *N*-(((2*S*,5*R*)-6-(Benzyloxy)-7-oxo-1,6-diazabicyclo[3.2.1]octan-2-yl)(imino)methyl)propionamide (**B2**) S7

2.13 *N*-(((2*S*,5*R*)-6-Hydroxy-7-oxo-1,6-diazabicyclo[3.2.1]octan-2-yl)(imino)methyl)propionamide (**C2**) S7

2.14 Sodium (2*S*,5*R*)-7-oxo-2-(N-propionylcarbamimidoyl)-1,6-diazabicyclo[3.2.1]octan-6-yl sulfate (**A2**) S7

2.15 3-Acetamido-*N*-(((2*S*,5*R*)-6-(benzyloxy)-7-oxo-1,6-diazabicyclo[3.2.1]octan-2-yl)(imino)methyl)propanamide (**B3**) S7

2.16 3-Acetamido-*N*-(((2*S*,5*R*)-6-hydroxy-7-oxo-1,6-diazabicyclo[3.2.1]octan-2-yl)(imino)methyl)propanamide (**C3**) S7

2.17 Sodium (2*S*,5*R*)-2-(N-(3-acetamidopropanoyl)carbamimidoyl)-7-oxo-1,6-diazabicyclo[3.2.1]octan-6-yl sulfate(**A3**) S7

2.18 *N*-(((2*S*,5*R*)-6-(Benzyloxy)-7-oxo-1,6-diazabicyclo[3.2.1]octan-2-yl)(imino)methyl)benzamide (**B4**) S8

2.19 *N*-(((2*S*,5*R*)-6-Hydroxy-7-oxo-1,6-diazabicyclo[3.2.1]octan-2-yl)(imino)methyl)benzamide(**C4**) S8

2.20 (2*S*,5*R*)-2-(*N*-Benzoylcarbamimidoyl)-7-oxo-1,6-diazabicyclo[3.2.1]octan-6-yl hydrogen sulfate (**A4**) S8

2.21 *N*-(((2*S*,5*R*)-6-(Benzyloxy)-7-oxo-1,6-diazabicyclo[3.2.1]octan-2-yl)(imino)methyl)-4-fluorobenzamide(**B5**) S8

2.22 4-Fluoro-*N*-(((2*S*,5*R*)-6-hydroxy-7-oxo-1,6-diazabicyclo[3.2.1]octan-2-yl)(imino)methyl)benzamide (**C5**) S8

2.23 (2*S*,5*R*)-2-(*N*-(4-Dluorobenzoyl)carbamimidoyl)-7-oxo-1,6-diazabicyclo[3.2.1]octan-6-yl hydrogen sulfate (**A5**) S8

2.24 *N*-(((2*S*,5*R*)-6-(Benzyloxy)-7-oxo-1,6-diazabicyclo[3.2.1]octan-2-yl)(imino)methyl)-4-(trifluoromethyl)benzamide (**B6**) S8

2.25 *N*-(((2*S*,5*R*)-6-Hydroxy-7-oxo-1,6-diazabicyclo[3.2.1]octan-2-yl)(imino)methyl)-4-(trifluoromethyl)benzamide (**C6**) S8

2.26 Sodium (2*S*,5*R*)-7-oxo-2-(*N*-(4-(trifluoromethyl)benzoyl)carbamimidoyl)-1,6-diazabicyclo[3.2.1]octan-6-yl sulfate (**A6**) S9

2.27 *N*-(((2*S*,5*R*)-6-(Benzyloxy)-7-oxo-1,6-diazabicyclo[3.2.1]octan-2-yl)(imino)methyl)-5-(trifluoromethyl)picolinamide (**B7**) S9

2.28 *N*-(((2*S*,5*R*)-6-Hydroxy-7-oxo-1,6-diazabicyclo[3.2.1]octan-2-yl)(imino)methyl)-5-(trifluoromethyl)picolinamide (**C7**) S9

2.29 Sodium (2*S*,5*R*)-7-oxo-2-(*N*-(5-(trifluoromethyl)picolinoyl)carbamimidoyl)-1,6-diazabicyclo[3.2.1]octan-6-yl sulfate (**A7**) S9

2.30 *N*-(((2*S*,5*R*)-6-(Benzyloxy)-7-oxo-1,6-diazabicyclo[3.2.1]octan-2-yl)(imino)methyl)-6-(trifluoromethyl)nicotinamide (**B8**) S9

2.31 *N*-(((2*S*,5*R*)-6-Hydroxy-7-oxo-1,6-diazabicyclo[3.2.1]octan-2-yl)(imino)methyl)-6-(trifluoromethyl)nicotinamide (**C8**) S9

2.32 Sodium (2*S*,5*R*)-7-oxo-2-(N-(6-(trifluoromethyl)nicotinoyl)carbamimidoyl)-1,6-diazabicyclo[3.2.1]octan-6-yl sulfate (**A8**) S9

2.33 *N*-(((2*S*,5*R*)-6-(Benzyloxy)-7-oxo-1,6-diazabicyclo[3.2.1]octan-2-yl)(imino)methyl)pyrimidine-5-carboxamide (**B9**) S10

2.34 *N*-(((2*S*,5*R*)-6-Hydroxy-7-oxo-1,6-diazabicyclo[3.2.1]octan-2-yl)(imino)methyl)pyrimidine-5-carboxamide (**C9**) S10

2.35 Sodium (2*S*,5*R*)-7-oxo-2-(*N*-(pyrimidine-5-carbonyl)carbamimidoyl)-1,6-diazabicyclo[3.2.1]octan-6-yl sulfate (**A9**) S10

2.36 *N*-(((2*S*,5*R*)-6-(Benzyloxy)-7-oxo-1,6-diazabicyclo[3.2.1]octan-2-yl)(imino)methyl)pyrimidine-4-carboxamide (**B10**) S10

2.37 *N*-(((2*S*,5*R*)-6-Hydroxy-7-oxo-1,6-diazabicyclo[3.2.1]octan-2-yl)(imino)methyl)pyrimidine-4-carboxamide (**C10**) S10

2.38 Sodium (2*S*,5*R*)-7-oxo-2-(*N*-(pyrimidine-4-carbonyl)carbamimidoyl)-1,6-diazabicyclo[3.2.1]octan-6-yl sulfate (**A10**) S10

2.39 *N*-(((2*S*,5*R*)-6-(Benzyloxy)-7-oxo-1,6-diazabicyclo[3.2.1]octan-2-yl)(imino)methyl)-2-(pyrimidin-2-yl)acetamide (**B11**) ... S10
2.40 *N*-(((2*S*,5*R*)-6-Hydroxy-7-oxo-1,6-diazabicyclo[3.2.1]octan-2-yl)(imino)methyl)-2-(pyrimidin-2-yl)acetamide (**C11**) ... S10
2.41 Sodium (2*S*,5*R*)-7-oxo-2-(*N*-(2-(pyrimidin-2-yl)acetyl)carbamimidoyl)-1,6-diazabicyclo[3.2.1]octan-6-yl sulfate (**A11**) ... S11
2.42 *N*-(((2*S*,5*R*)-6-(Benzyloxy)-7-oxo-1,6-diazabicyclo[3.2.1]octan-2-yl)(imino)methyl)-3-(pyridin-2-yl)propanamide (**B12**) ... S11
2.43 Sodium (2*S*,5*R*)-7-oxo-2-(*N*-(3-(pyridin-2-yl)propanoyl)carbamimidoyl)-1,6-diazabicyclo[3.2.1]octan-6-yl sulfate(**A12**) ... S11
2.44 *N*-(((2*S*,5*R*)-6-(Benzyloxy)-7-oxo-1,6-diazabicyclo[3.2.1]octan-2-yl)(imino)methyl)furan-2-carboxamide (**B13**) ... S11
2.45 *N*-(((2*S*,5*R*)-6-^{h}ydroxy-7-oxo-1,6-diazabicyclo[3.2.1]octan-2-yl)(imino)methyl)furan-2-carboxamide (**C13**) ... S11
2.46 (2*S*,5*R*)-2-(*N*-(Furan-2-carbonyl)carbamimidoyl)-7-oxo-1,6-diazabicyclo[3.2.1]octan-6-yl hydrogen sulfate (**A13**) ... S11
2.47 *N*-(((2*S*,5*R*)-6-(Benzyloxy)-7-oxo-1,6-diazabicyclo[3.2.1]octan-2-yl)(imino)methyl)oxazole-5-carboxamide (**B14**) ... S11
2.48 *N*-(((2*S*,5*R*)-6-Hydroxy-7-oxo-1,6-diazabicyclo[3.2.1]octan-2-yl)(imino)methyl)oxazole-5-carboxamide (**C14**) ... S11
2.49 Sodium (2*S*,5*R*)-2-(*N*-(oxazole-5-carbonyl)carbamimidoyl)-7-oxo-1,6-diazabicyclo[3.2.1]octan-6-yl sulfate (**A14**) ... S12
2.50 *N*-(((2*S*,5*R*)-6-(Benzyloxy)-7-oxo-1,6-diazabicyclo[3.2.1]octan-2-yl)(imino)methyl)oxazole-4-carboxamide (**B15**) ... S12
2.51 *N*-(((2*S*,5*R*)-6-Hydroxy-7-oxo-1,6-diazabicyclo[3.2.1]octan-2-yl)(imino)methyl)oxazole-4-carboxamide (**C15**) ... S12
2.52 Sodium (2*S*,5*R*)-2-(*N*-(oxazole-4-carbonyl)carbamimidoyl)-7-oxo-1,6-diazabicyclo[3.2.1]octan-6-yl sulfate (**A15**) ... S12
2.53 *N*-(((2*S*,5*R*)-6-(Benzyloxy)-7-oxo-1,6-diazabicyclo[3.2.1]octan-2-yl)(imino)methyl)cyclohexanecarboxamide (**B16**) ... S12
2.54 *N*-(((2*S*,5*R*)-6-Hydroxy-7-oxo-1,6-diazabicyclo[3.2.1]octan-2-yl)(imino)methyl)cyclohexanecarboxamide (**C16**) ... S12
2.55 Sodium (2*S*,5*R*)-2-(*N*-(cyclohexanecarbonyl)carbamimidoyl)-7-oxo-1,6-diazabicyclo[3.2.1]octan-6-yl sulfate (**A16**) ... S12
2.56 4-Acetamido-*N*-(((2*S*,5*R*)-6-(benzyloxy)-7-oxo-1,6-diazabicyclo[3.2.1]octan-2-yl)(imino)methyl)cyclohexane-1-carboxamide (**B17**) ... S12
2.57 4-Acetamido-*N*-(((2*S*,5*R*)-6-hydroxy-7-oxo-1,6-diazabicyclo[3.2.1]octan-2-yl)(imino)methyl)cyclohexane-1-carboxamide (**C17**) ... S12
2.58 Sodium (2*S*,5*R*)-2-(*N*-(4-acetamidocyclohexane-1-carbonyl)carbamimidoyl)-7-oxo-1,6-diazabicyclo[3.2.1]octan-6-yl sulfate (**A17**) ... S13
2.59 *tert*-Butyl 4-((((2*S*,5*R*)-6-(benzyloxy)-7-oxo-1,6-diazabicyclo[3.2.1]octan-2-yl)(imino)methyl)carbamoyl)piperidine-1-carboxylate (**B18**) ... S13
2.60 *tert*-Butyl 4-((((2*S*,5*R*)-6-hydroxy-7-oxo-1,6-diazabicyclo[3.2.1]octan-2-yl)(imino)methyl)carbamoyl)piperidine-1-carboxylate (**C18**) ... S13
2.61 Sodium (2*S*,5*R*)-7-oxo-2-(*N*-(piperidine-4-carbonyl)carbamimidoyl)-1,6-diazabicyclo[3.2.1]octan-6-yl sulfate (**A18**) ... S13
2.62 (3*R*)-1-Acetyl-*N*-(((2*S*,5*R*)-6-(benzyloxy)-7-oxo-1,6-diazabicyclo[3.2.1]octan-2-yl)(imino)methyl)piperidine-3-carboxamide (**B19**) ... S13
2.63 (3*R*)-1-Acetyl-*N*-(((2*S*,5*R*)-6-hydroxy-7-oxo-1,6-diazabicyclo[3.2.1]octan-2-yl)(imino)methyl)piperidine-3-carboxamide (**C19**) ... S13
2.64 Sodium (2*S*,5*R*)-2-(*N*-((*R*)-1-acetylpiperidine-3-carbonyl)carbamimidoyl)-7-oxo-1,6-diazabicyclo[3.2.1]octan-6-yl sulfate (**A19**) ... S14
2.65 (3*S*)-1-Acetyl-*N*-(((2*S*,5*R*)-6-(benzyloxy)-7-oxo-1,6-diazabicyclo[3.2.1]octan-2-yl)(imino)methyl)piperidine-3-carboxamide (**B20**) ... S14
2.66 (3*S*)-1-Acetyl-*N*-(((2*S*,5*R*)-6-hydroxy-7-oxo-1,6-diazabicyclo[3.2.1]octan-2-yl)(imino)methyl)piperidine-3-carboxamide (**C20**) ... S14
2.67 Sodium (2*S*,5*R*)-2-(*N*-((*S*)-1-acetylpiperidine-3-carbonyl)carbamimidoyl)-7-oxo-1,6-diazabicyclo[3.2.1]octan-6-yl sulfate (**A20**) ... S14
2.68 *N*-(((2*S*,5*R*)-6-(Benzyloxy)-7-oxo-1,6-diazabicyclo[3.2.1]octan-2-yl)(imino)methyl)-1-methylpiperidine-4-carboxamide (**B21**) ... S14
2.69 *N*-(((2*S*,5*R*)-6-Hydroxy-7-oxo-1,6-diazabicyclo[3.2.1]octan-2-yl)(imino)methyl)-1-methylpiperidine-4-carboxamide (**C21**) ... S14
2.70 Sodium (2*S*,5*R*)-2-(*N*-(1-methylpiperidine-4-carbonyl)carbamimidoyl)-7-oxo-1,6-diazabicyclo[3.2.1]octan-6-yl sulfate (**A21**) ... S14
2.71 (2*R*)-1-Acetyl-*N*-(((2*S*,5*R*)-6-((*tert*-butyldimethylsilyl)oxy)-7-oxo-1,6-diazabicyclo[3.2.1]octan-2-yl)(imino)methyl)piperidine-2-carboxamide (**B22**) ... S14
2.72 (2*R*)-1-Acetyl-*N*-(((2*S*,5*R*)-6-hydroxy-7-oxo-1,6-diazabicyclo[3.2.1]octan-2-yl)(imino)methyl)piperidine-2-carboxamide(**C22**) ... S15

2.73 Sodium (2*S*,5*R*)-2-(*N*-((*R*)-1-acetylpiperidine-2-carbonyl)carbamimidoyl)-7-oxo-1,6-diazabicyclo[3.2.1]octan-6-yl sulfate (**A22**) S15
2.74 *tert*-Butyl(2-((((2*S*,5*R*)-6-((*tert*-butyldimethylsilyl)oxy)-7-oxo-1,6-diazabicyclo[3.2.1]octan-2-yl)(imino)methyl)carbamoyl)thiazol-4-yl)carbamate (**B23**) S15
2.75 *tert*-Butyl(2-((((2*S*,5*R*)-6-hydroxy-7-oxo-1,6-diazabicyclo[3.2.1]octan-2-yl)(imino)methyl)carbamoyl)thiazol-4-yl)carbamate (**C23**) S15
2.76 Sodium (2*S*,5*R*)-2-(*N*-(4-aminothiazole-2-carbonyl)carbamimidoyl)-7-oxo-1,6-diazabicyclo[3.2.1]octan-6-yl sulfate(**A23**) S15
3 Determination of antibacterial activity S16
4 NMR spectra of final compounds **A1**–**23** S17
References S39

1. General methods and materials

All ^{1}H and ^{19}F NMR spectra were recorded on a Bruker AVANCE NEO 400 NMR operating at 400 MHz for ^{1}H, and 376 MHz for ^{19}F, respectively. The NMR data are recorded as chemical shifts relative to tetramethylsilane (TMS) as internal standard. NMR spectra were run either in $CDCl_3$ containing 0.05% TMS, CD_3OD containing 0.05% TMS, D_2O or DMSO-d_6 containing 0.03% TMS. Chemical shifts (δ) are given in parts per million (ppm) and coupling constant (*J* values) are given in hertz (Hz). Signal multiplicities are reported as follows: s, singlet; brs, broad singlet; d, doublet; t, triplet and m, multiplet. Preparative HPLC was performed on an Agilent 1260 Infinity II System on Agilent 10 prep-C18 250 × 21.2 mm column, using an acetonitrile/aqueous 0.1% trifluoroacetic acid gradient, or an acetonitrile/aqueous 0.1% formic acid gradient, or an acetonitrile/water at 22 °C. Mass spectra were performed on an Agilent 1260II-6125 Separation Module using either ES^- or ES^+ ionization modes. Column chromatography was performed using Qingdao Inc. Silica Gel, CC Grade (230–400 mesh). Commercial solvents and reagents were generally used without further purification. All products were dried before characterization and use in subsequent synthetic steps.

2. Experimental procedures

2.1 (2*S*,5*R*)-*6-(Benzyloxy)-7-oxo-1,6-diazabicyclo[3.2.1]octane-2-carbonitrile* ***(7):*** trifluoroacetic anhydride (TFAA, 0.3 g, 1.4 mmol) was added to a mixture of **6** (0.2 g, 0.7 mmol) and TEA (0.7 g, 7.0 mmol) in CH_2Cl_2 (5 mL) at 0 °C. The resulting reaction mixture was heated at 35 °C for 3 hours, and then concentrated under reduced pressure. The residue was extracted with ethyl acetate, washed with water, brine, dried over Na_2SO_4, and filtrated. The filtrate was concentrated to give a residue, which was further purified by column chromatography eluting with 30% ethyl acetate in hexane to give the title compound **7** (0.17 g, 64%) as a brown solid. ^{1}H NMR (400 MHz, DMSO-d_6): *δ* 1.81-1.91(m, 2H), 1.92-2.00 (m, 2H), 3.10 (d, *J* = 11.7 Hz, 1H), 3.21 (d, *J* = 12.3 Hz, 1H), 3.74 (s, 1H), 4.58 (d, *J* = 6.6 Hz, 1H), 4.93 (d, *J* = 11.6 Hz, 1H), 4.96 (d, *J* = 11.6 Hz, 1H), 7.36-7.43 (m, 3H), 7.44-7.48 (m, 3H). LC-MS $[M+H]^+$ *m/z* 258.1 (calcd for $C_{14}H_{16}N_3O_2$, 257.12).

2.2 (2*S*,5*R*)-*6-(benzyloxy)-7-oxo-1,6-diazabicyclo[3.2.1]octane-2-carboximidamide (**1**):* $AlMe_3$ in n-hexane (2 N, 9.0 mL, 18.0 mmol) and NH_4Cl (0.96 g, 18.0 mmol) were added to a solution of **7** (3.08 g, 15.0 mmol) in anhydrous CH_2Cl_2 (45 mL) at 0 °C. The reaction mixture was stirred at room temperature overnight, cooled to 0 °C, quenched by addition of silica gel (8 g) and methanol (8 mL). The resulting mixture was stirred at room temperature for 20 min, filtered off, rinsed with 10% methanol in CH_2Cl_2 (2×30 mL). The filtrate was concentrated and purified by flash column chromatography using 2-5% MeOH in CH_2Cl_2 to give the title compound **1** (1.45 g, 44%) as a white solid. ^{1}H NMR (400 MHz, DMSO-d_6): *δ* 1.61-1.79 (m, 2H), 1.80-1.92 (m, 2H), 2.77-2.95 (m, 2H), 3.12-3.21 (s, 1H), 3.80-3.92 (m, 1H), 4.09-4.15 (m, 1H), 4.74-4.84 (m, 2H), 6.52-6.66 (s, 2H), 7.33-7.42 (m, 3H), 7.45-7.54 (m, 2H). LC-MS $[M+H]^+$ *m/z* 275.1 (calcd for $C_{14}H_{18}N_4O_2$, 274.14).

2.3 (2*S*,5*R*)-*6-Hydroxy-7-oxo-1,6-diazabicyclo[3.2.1]octane-2-carbonitrile (**8**):* Wet 10%Pd/C (720 mg) was added to a solution of compound **7** (3.5 g, 13.6 mmol) in EtOAc and CH_2Cl_2 (2:1, 15 mL), and then hydrogenated at room temperature under 45 psi pressure for 2 hours. After completion of reaction, the catalyst was removed by celite filtration and washed with EtOAc. The filtrate was concentrated to obtain the pale yellow compound **8** (2.1 g, 95%) as a crude product, which was used without purification for further reactions. ^{1}H NMR (400 MHz, $CDCl_3$): *δ* 1.68-1.70 (m, 1H), 1.87-1.93 (m, 2H), 2.05-2.18 (m, 1H), 3.14-3.25 (m, 2H), 4.01-4.14 (m, 1H), 5.21 (brs, 1H). LC-MS $[M+H]^+$ *m/z* 168.1 (calcd for $C_7H_9N_3O_2$, 167.07).

2.4 (2*S*,5*R*)-*6-((tert-Butyldimethylsilyl)oxy)-7-oxo-1,6-diazabicyclo[3.2.1]octane-2-carbonitrile (**10**): tert-Butyl*dimethylsilyl chloride (**9**, TBSCl, 2 g, 13.5 mmol) was added to a stirred solution of compound **8** (1.5 g, 9 mmol) and imidazole (1.2 g, 18 mmol) in CH_2Cl_2 (20 mL). The reaction mixture was stirred at room temperature overnight. The solids formed were filtered and the filtrate was washed with 0.1 N HCl followed by water and brine. The organic layer was dried over $MgSO_4$ and concentrated to furnish the crude product which was purified by column chromatography using CH_2Cl_2 as a solvent to give the title compound **10** (1.16 g, 46%) as a white solid. ^{1}H NMR (400 MHz, $CDCl_3$): δ 0.00 (s, 3H), 0.06 (s, 3H), 0.78 (s, 9H), 1.69-1.76 (m, 2H), 2.00-2.13 (m, 2H), 3.00 (d, *J* = 11.5 Hz, 1H), 3.19 (d, *J* = 12.2 Hz, 1H), 3.46 (s, 1H), 4.17 (d, *J* = 7.2 Hz, 1H). LC-MS $[M+H]^+$ *m/z* 282.2 (calcd for $C_{13}H_{23}N_3O_2Si$, 281.16).

2.5 (2*S*,5*R*)-*6-((tert-Butyldimethylsilyl)oxy)-7-oxo-1,6-diazabicyclo[3.2.1]octane-2-carboximidamide (**2**):* Trimethylaluminum (2 M in hexane, 2.9 mL, 5.8 mmol) was added dropwise to a suspension of ammonium chloride (358 mg, 5.87 mmol) in CH_2Cl_2 (10 mL) at room temperature. The suspension was further stirred for 30 minutes followed by the dropwise addition of compound **10** (1.1 g, 3.9 mmol) dissolved in CH_2Cl_2 (10 mL). The reaction mixture was stirred overnight at room temperature while the progress of reaction was monitored by LCMS. Another portion of NH_4Cl (358 mg, 5.87 mmol) and trimethylaluminum (2.9 mL, 5.8 mmol) was added and the reaction mixture was further stirred at room temperature for 24 hours. Upon completion of the reaction MeOH (50 mL) was added dropwise to quench the unreacted trimethylaluminum. The bulk of solid formed was filtered and the filter cake was washed with MeOH. The filtrate was concentrated to afford the white solid which was purified by column chromatography using CH_2Cl_2 containing 2–4% MeOH to give the title product **2** (263 mg, 23%) as a white solid. ^{1}H NMR (400 MHz, $CDCl_3$): δ 0.00 (s, 6H), 0.77 (s, 9H), 1.62-1.66 (m, 2H), 1.73-1.78 (m, 1H), 1.83-1.87 (m, 1H), 2.80 (dd, *J* = 11.2 Hz, 3.18Hz, 1H), 2.95 (t, *J* = 11.2 Hz, 1H), 3.64-3.72 (m, 1H), 3.80 (d, *J* = 2.1 Hz, 1H), 5.76 (br s, 2H). LC-MS $[M+H]^+$ *m/z* 299.2 (calcd for $C_{13}H_{26}N_4O_2Si$, 298.18).

2.6 *(R)-1-Acetylpiperidine-3-carboxylate (**3**):* Ac_2O (1.4 g, 14.3 mmol) was added to a solution of **11** (2.0 g, 12.7 mmol) in CH_2Cl_2 (20 mL), and the mixture was stirred at room temperature for 24 hours. The reaction mixture was concentrated to give a residue, which was further purified by silica gel column chromatography eluting with 50% ethyl acetate in petroleum ether to give the intermediate acetylated compound (2.41 g, 95%) as an oil. ^{1}H NMR (400 MHz, $CDCl_3$): δ 1.25(t, *J* = 7.1 Hz,1.5H), 1.28(t, *J* = 7.1 Hz, 1.5H), 1.44-1.55 (m, 1H), 1.64-1.73 (m, 1H), 1.76-1.86 (m, 1H), 1.97-2.07 (m, 1H), 2.09 (s, 1.5H), 2.14 (s, 1.5H), 2.39-2.52 (m, 1H), 2.80-2.87 (m, 0.5H), 3.03-3.14 (m, 1H), 3.43-3.50 (m, 0.5H), 3.68-3.77 (m, 1H), 3.94-4.01 (m, 0.5H), 4.10-4.20 (m, 2H), 4.59-4.65 (m, 0.5H). In a next step, NaOH (2 N, 6 mL, 12 mmol) was added to a solution of the intermediate obtained above (1.2 g, 6.0 mmol) in THF (8 mL) at 0 °C, and stirred at 0 °C for 2 hours. The reaction mixture was concentrated at 13 °C to remove THF giving a residue, which was washed by CH_2Cl_2 (10 × 2 mL), the aqueous layer was acidified to pH ≈ 4 by 3 N HCl at 0 °C, and lyophilized to give a residue,which was extracted with CH_2Cl_2, the CH_2Cl_2 layer was concentrated to give the title compound **3** (1.04 g, quantitative yield) as a solid. ^{1}H NMR (400 MHz, DMSO-d_6): δ 1.24-1.43 (m, 1H), 1.48-1.56 (m, 1H), 1.58-1.68 (m, 1H), 1.81-1.89 (m, 1H), 1.97 (s, 1.5H), 2.00 (s, 1.5H), 2.06-2.15 (m, 0.5H), 2.36-2.33 (m, 0.5H), 2.53-2.60 (m, 0.5H), 2.87-2.99 (m, 1H), 3.24-3.31 (m, 0.5H), 3.62-3.71 (m, 1H), 3.75-3.81 (m, 0.5H), 4.35-4.42 (m, 0.5H). LC-MS $[M+H]^+$ *m/z* 172.1 (calcd for $C_8H_{13}NO_3$, 171.09).

***2.7** (S)-1-Acetylpiperidine-3-carboxylate (**4**):* Ac_2O (1.4 g, 14.3 mmol) was added to a solution of **12** (2.0 g, 12.7 mmol) in CH_2Cl_2 (20 mL), and stirred at room temperature for 24 hours. The reaction mixture was concentrated to give a residue, which was further purified by silica gel column chromatography eluting with 50% ethyl acetate in petroleum ether to give the intermediate compound (2.51 g, 99%) as an oil. ^1H NMR (400 MHz, CD_3Cl): δ 1.25(t, J = 7.1 Hz, 1.5H), 1.28 (t, J = 7.1 Hz, 1.5H), 1.41-1.55 (m, 1H), 1.64-1.73 (m, 1H), 1.76-1.86 (m, 1H), 1.97-2.07 (m, 1H), 2.09 (s, 1.5H), 2.14 (s, 1.5H), 2.39-2.52 (m, 1H), 2.80-2.87 (m, 0.5H), 3.03-3.14 (m, 1H), 3.43-3.50 (m, 0.5H), 3.68-3.77 (m, 1H), 3.94-4.01 (m, 0.5H), 4.10-4.20 (m, 2H), 4.59-4.65 (m, 0.5H). In the next step, NaOH (2 N, 6 mL, 12 mmol) was added to a solution of the intermediate compound obtained above (1.2 g, 6.0 mmol) in THF (8 mL) at 0 °C, and the mixture stirred at room temperature for 2 hours. The reaction mixture was concentrated to remove THF, water (5 mL) was added, washed by CH_2Cl_2 (10 × 2 mL). The aqueous layer was acidified to pH ≈ 4 by 3 N HCl at 0 °C, and lyophilized to give a residue,which was extracted with CH_2Cl_2, the CH_2Cl_2 layer was concentrated to give the title compound **4** (0.88 g, 85%) as a solid. ^1H NMR (400 MHz, $CDCl_3$): δ 1.44-1.57(m, 1H),1.65-1.73 (m, 0.5H), 1.74-1.90 (m, 1.5H), 1.93-2.03 (m, 1H), 2.09 (s, 1.5H), 2.15 (s, 1.5H), 2.41-2.52 (m, 1H), 3.13-3.20 (m, 0.5H), 3.24-3.32 (m, 0.5H), 3.38-3.57 (m, 1.5H), 3.71-3.77 (m, 0.5H), 3.88-3.96 (m, 0.5H), 4.01-4.08 (m, 0.5H), 8.12 (brs, 1H). LC-MS $[M+H]^+$ *m/z* 172.1 (calcd for $C_8H_{13}NO_3$, 171.09).

***2.8** (S)-1-Acetylpiperidine-2-carboxylic acid (**5**):* (*R*)-piperidine-2-carboxylic acid (**13**, 1 g, 7.7 mmol) was added to a suspension of acetic anhydride (3 mL, 116 mmol) in water (2 mL), and stirred at room temperature for 3 hours. EtOH (100 mL) was added to the reaction mixture and concentrated to afford a slurry which was treated with EtOAc and petroleum ether (30/30 mL) to furnish a white solid. The solid was collected by filtration and dried in air to give the title compound **5** (0.9 g, 68%) as a solid. ^1H NMR (400 MHz, DMSO-d_6): δ 1.24-1.55 (m, 5H), 1.88 (s, 2H), 1.94 (s, 1H), 2.19-2.31(m, 1.2H), 2.64-2.73 (m, 0.8H), 3.34-3.44 (m, 0.6H), 4.06-4.10 (m, 0.6H), 4.16-4.22 (m, 0.4H), 4.76-4.80 (m, 0.4H). LC-MS $[M+H]^+$ *m/z* 172.1 (calcd for $C_8H_{13}NO_3$, 171.09).

***2.9** N-((*(2*S*,5*R*)*-6-(benzyloxy)-7-oxo-1,6-diazabicyclo[3.2.1]octan-2-yl)(imino)methyl)acetamide (**B1**):* Acetyl chloride (0.05 mL, 0.70 mmol) was added to a solution of **1** (150 mg, 0.54 mmol) and TEA (0.17 mL, 1.21 mmol) in CH_2Cl_2 (10 mL) at room temperature, and then stirred at room temperature under argon overnight. The reaction mixture was diluted with CH_2Cl_2, washed with water, saturated NH_4Cl, dried over Na_2SO_4, and filtrated. The organic layer was concentrated to give a residue, which was purified by silica gel column chromatography eluting with 30% ethyl acetate in hexane to give the title compound **B1** (167 mg, 97%) as an oil. ^1H NMR (400 MHz, $CDCl_3$): δ 1.73-1.83 (m, 1H), 2.01-2.08 (m, 1H), 2.10 (s, 3H), 2.14-2.32 (m, 2H), 3.38-3.50 (m, 1H), 3.70-3.82 (m, 1H), 4.02-4.09 (m, 1H), 4.85 (d, J = 10.8 Hz, 1H), 4.92 (d, J = 10.8 Hz, 1H), 5.48 (s, 2H), 5.75-5.80 (m, 1H), 7.39-7.44 (m, 5H). LC-MS $[M+H]^+$ *m/z* 339.2 (calcd for $C_{16}H_{20}N4O_3$, 316.15).

***2.10** N-((*(2*S*,5*R*)*-6-hydroxy-7-oxo-1,6-diazabicyclo[3.2.1]octan-2-yl)(imino)methyl)acetamide (**C1**):* 10% Pd/C (wet, 55% water w/w, 20 mg) was added to a solution of compound **B1** (100 mg, 0.32 mmol) in EtOAc (5 mL). The mixture was stirred under H_2 (balloon) at room temperature overnight, filtered through a pad of celite, and rinsed with EtOAc. The filtrate was concentrated to give the title compound **C1** (60 mg, 81%) as a white solid, that was directly used in the next step without further purification. LC-MS $[M+Na]^+$ *m/z* 249.1 (calcd for $C_9H_{14}NO_3$, 226.11).

2.11 (2*S*,5*R*)*-2-(N-Acetylcarbamimidoyl)-7-oxo-1,6-diazabicyclo[3.2.1]octan-6-yl hydrogen sulfate (**A1**):* A mixture of compound **C1** (60 mg, 0.26 mmol), $SO_3 \cdot NMe_3$ complex (183 mg, 1.32 mmol) and TEA (1 mL, 7.22 mmol) in THF/water (5:5 mL) was stirred at room temperature overnight. The reaction mixture was concentrated under reduced pressure to provide a residue, which was purified by preparative HPLC on an Agilent 10 prep-C18 250 × 21.2 mm column and lyophilized to give **A1** (10 mg, 12%) as a white solid. ^1H NMR (400 MHz, CD_3OD): δ 1.89-2.01 (m, 2H), 2.07 (s, 3H), 2.08-2.32 (m, 2H), 3.54 (dd, J = 12.8, 11.2 Hz, 1H), 3.88-4.03 (m, 2H), 5.62 (d, J = 4.3 Hz, 1H). LC-MS $[M-H]^-$ *m/z* 305.1 (calcd for $C_9H_{14}N_4O_6S$, 306.06).

2.12 *N-((*(2*S*,5*R*)*-6-(benzyloxy)-7-oxo-1,6-diazabicyclo[3.2.1]octan-2-yl)(imino)methyl)propionamide (**B2**):* Propionic acid (27 mg, 0.36 mmol) was added to a solution of **1** (100 mg, 0.36 mmol), DAMP (67 mg, 0.55 mmol) and DCC (114 mg, 0.55 mmol) in CH_2Cl_2 (10 mL) at room temperature, and then stirred at room temperature under argon overnight. The reaction mixture was diluted with CH_2Cl_2, washed with water, saturated NH_4Cl, dried over Na_2SO_4, and filtrated. The organic layer was concentrated to give a residue, which was purified by silica gel column chromatography eluting with 30% ethyl acetate in hexane to give the title compound **B2** (100 mg, 84%) as an oil. ^{1}H NMR (400 MHz, $CDCl_3$): δ 1.36 (t, *J* = 7.3 Hz, 3H), 1.65-1.83 (m, 2H), 1.87-1.96 (m, 1H), 2.01-2.13 (m, 1H), 2.36 (q, *J* = 7.3 Hz, 2H), 3.37-3.47 (m, 1H), 3.77-3.84 (m, 1H), 4.02-4.09 (m, 1H), 4.85 (d, *J* = 10.5 Hz, 1H), 4.93 (d, *J* = 10.5 Hz, 1H), 5.22 (br s, 2H), 5.78-5.83 (m, 1H), 7.35-7.43 (3, 5H). LC-MS $[M+Na]^+$ *m/z* 353.2 (calcd for $C_{17}H_{22}N_4O_3$, 330.17).

2.13 *N-((*(2*S*,5*R*)*-6-Hydroxy-7-oxo-1,6-diazabicyclo[3.2.1]octan-2-yl)(imino)methyl)propionamide (**C2**):* Compound **C2** (80 mg, quantitative) as an oil was prepared from **B2** (100 mg, 0.30 mmol) by using the procedure described for **C1**. ^{1}H NMR (400 MHz, CD_3OD): δ 1.13 (t, *J* = 7.3 Hz, 3H), 1.82-1.93 (m, 2H), 2.08-2.18 (m, 2H), 2.39-2.57 (m, 2H), 3.37-3.45 (m, 1H), 3.87-3.94 (m, 1H), 4.02-4.14 (m, 1H), 5.75-5.79 (m,1H). LC-MS $[M+Na]^+$ *m/z* 263.1 (calcd for $C_{10}H_{16}N_4O_3$, 240.12).

2.14 *Sodium* (2*S*,5*R*)*-7-oxo-2-(N-propionylcarbamimidoyl)-1,6-diazabicyclo[3.2.1]octan-6-yl sulfate (**A2**):* Compound **A2** as white solid was prepared from **C2** (105 mg, 0.3 mmol) and $SO_3{\cdot}NMe_3$ complex (90 mg, 0.65 mmol) by using the procedure described for **A1**. After HPLC purification the compound was ion exchanged by Dowex-50wx Na^+ resin using water as an eluent to give **A2** (30 mg, 31% in two steps). ^{1}H NMR (400 MHz, D_2O): δ 1.00 (t, *J* = 7.2 Hz, 3H), 1.71-1.81 (m, 1H), 1.90-2.00 (m, 1H), 2.03-2.14 (m, 2H), 2.42 (q, *J* = 7.2 Hz, 2H), 3.49 (t, *J* = 11.7 Hz, 1H), 3.92-4.05 (m,2H), 5.67 (d, *J* = 3.9 Hz, 1H). LC-MS $[M-Na]^-$ *m/z* 319.1 (calcd for $C_{10}H_{15}NaN_4O_6S$, 342.07).

2.15 *3-Acetamido-N-((*(2*S*,5*R*)*-6-(benzyloxy)-7-oxo-1,6-diazabicyclo[3.2.1]octan-2-yl)(imino)methyl)propanamide (**B3**)*: 3-Acetamidopropanoic acid (157 mg, 1.98 mmol) was added to a solution of **1** (220 mg, 0.80 mmol), HATU (457 mg, 1.34 mmol) and DIPEA (0.44 mL, 2.57 mmol) in CH_2Cl_2/DMF (each 3 mL) at room temperature, and then stirred at room temperature for 24 hours. The reaction mixture was diluted with EtOAc, washed with saturated $NaHCO_3$, water, brine, dried over Na_2SO_4, and filtrated. The organic layer was concentrated to give a residue, which was purified by silica gel column chromatography eluting with 5% MeOH in $CH_2Cl_2$to give the title compound **B3** (300 mg, 96%) as an oil. ^{1}H NMR (400 MHz, DMSO-*d*): δ 1.67-2.00 (m, 7H), 2.44-2.53 (m, 2H), 3.16-3.27 (m, 3H), 3.73-3.83 (m, 1H), 3.88-3.96 (m, 1H), 4.83 (s, 2H), 5.66 (s, 1H), 6.76 (s, 2H), 7.35-7.40 (m, 3H), 7.46-7.52 (m, 2H), 7.90 (s, 1H). LC-MS $[M+H]^+$ *m/z* 388.2 (calcd for $C_{19}H_{25}N_5O_4$, 387.19).

2.16 *3-Acetamido-N-((*(2*S*,5*R*)*-6-hydroxy-7-oxo-1,6-diazabicyclo[3.2.1]octan-2-yl)(imino)methyl)propanamide (**C3**):* Compound **C3** (124 mg, 70%) as white solid was prepared from **B3** (230 mg, 0.59 mmol) by using the procedure described for **C1**. ^{1}H NMR (400 MHz, DMSO-d_6): δ 1.70-1.76 (m, 2H), 1.77 (s, 3H), 1.83-1.91 (m, 1H), 1.96-2.02 (m, 1H), 2.47-2.51 (m, 2H), 3.15-3.18 (m, 1H), 3.18-3.26 (m 2H), 3.65-3.71 (m, 1H), 3.92-4.00 (m, 1H), 5.67 (s, 1H), 6.50 (s, 2H), 7.90 (s, 1H), 9.30 (s, 1H). LC-MS $[M+H]^+$ *m/z* 298.2 (calcd for $C_{12}H_{19}N_5O_4$, 297.14).

2.17 *Sodium* (2*S*,5*R*)*-2-(N-(3-acetamidopropanoyl)carbamimidoyl)-7-oxo-1,6-diazabicyclo[3.2.1]octan-6-yl sulfate (**A3**):* A mixture of compound **C3** (122 mg, 0.41 mmol), $SO_3{\cdot}$pyridine (330 mg, 2.08 mmol) in pyridine (5 mL) was stirred at room temperature overnight. Another portion of $SO_3{\cdot}$pyridine (100 mg) was added to the reaction mixture, and stirring continued at room temperature for additional 3 days. The reaction mixture was concentrated to dryness under reduced pressure to give a residue, which was dissolved in water (20 mL) and lyophilized to give a solid. The solid was purified by preparative HPLC on an Agilent 10 prep-C18 250 × 21.2 mm column and lyophilized to give the desired compound, which was further purified by Dowex-50wx Na^+ resin, using water as an elution solvent to give **A3** (23 mg, 15% in two steps) as a white solid. ^{1}H NMR (400 MHz, D_2O): δ 1.61-1.71 (m, 1H), 1.78 (s, 3H), 1.82-1.91 (m, 1H), 1.93-2.05 (m, 2H), 2.54 (t, *J* = 6.3 Hz, 2H), 3.21-3.33 (m, 2H), 3.40 (t, *J* = 13.4 Hz, 1H), 3.85-3.94 (m, 2H), 5.57 (d, *J* = 3.5 Hz, 1H). LC-MS $[M-Na]^-$ *m/z* 376.1 (calcd for $C_{12}H_{18}NaN_5O_7S$, 399.08).

2.18 *N-((*(2*S*,5*R*)*-6-(Benzyloxy)-7-oxo-1,6-diazabicyclo[3.2.1]octan-2-yl)(imino)methyl)benzamide (**B4**)*: Compound **B4** (140 mg, 67%) was obtained as an oil from benzoic acid (67 mg, 0.55 mmol)and **1** (100 mg, 0.36 mmol) by using the procedure described for **B2**. ^{1}H NMR (400 MHz, $CDCl_3$): *δ* 1.77-1.93 (m, 2H), 2.05-2.36 (m, 2H), 3.24-3.35 (m, 1H), 4.07-4.25 (m, 2H), 4.66-4.83 (m, 2H), 5.39(s, 2H),7.27-7.42(m, 10H). LC-MS [M+Na]$^+$ *m/z* 401.2 (calcd for $C_{21}H_{25}N_4O_3$, 378.17).

2.19 *N-((*(2*S*,5*R*)*-6-Hydroxy-7-oxo-1,6-diazabicyclo[3.2.1]octan-2-yl)(imino)methyl)benzamide(**C4**):* Compound **C4** (100 mg, 93%) was obtained as white solid from **B4** (140 mg, 0.37 mmol) by using the procedure described for **C1**. ^{1}H NMR (400 MHz, CD_3OD): *δ* 1.57-1.63 (m, 1H), 1.70-1.77 (m, 1H), 2.01-2.10 (m, 2H), 3.28-3.40 (m, 1H), 3.89-4.12 (m, 2H), 5.59（s, 1H), 7.36-7.43(m, 5H). LC-MS [M+Na]$^+$ *m/z* 311.1 (calcd for $C_{14}H_{16}N_4O_3$, 288.12).

2.20 (2*S*,5*R*)*-2-(N-Benzoylcarbamimidoyl)-7-oxo-1,6-diazabicyclo[3.2.1]octan-6-yl hydrogen sulfate (**A4**):* Compound **A4** (30 mg, 24%) as white solid was prepared from **C4** (100 mg, 0.33 mmol) by using the procedure described for **A1**. ^{1}H NMR (400 MHz, DMSO-*d6*): *δ* 1.82-2.08 (m, 4H), 3.17-3.31 (m, 1H), 3.71-3.85 (m, 1H), 3.98-4.07 (m, 1H), 5.57-5.69 (m, 1H), 6.54 (br s, 2H), 7.41-7.54 (m, 5H). LC-MS [M-H]$^-$ *m/z* 367.1 (calcd for $C_{10}H_{16}N_4O_6S$, 368.08).

2.21 *N-((*(2*S*,5*R*)*-6-(Benzyloxy)-7-oxo-1,6-diazabicyclo[3.2.1]octan-2-yl)(imino)methyl)-4-fluorobenzamide (**B5**):* Compound **B4** (287 mg, 66%) was obtained as white solid from 4-fluorobenzoic acid (155 mg, 1.09 mmol) and **1** (200 mg, 0.73 mmol) by using the procedure described for **B2**. ^{1}H NMR(400 MHz, CD_3OD): *δ* 1.80-1.92 (m, 2H), 1.98-2.15 (m, 2H), 3.01-3.18 (m, 1H), 3.30-3.55 (m, 1H), 3.91-3.98 (m, 1H), 4.78 (s, 2H), 5.41-5.56 (m, 1H), 7.09-7.14 (m, 2H), 7.21-7.26 (m, 2H). 7.27-7.34 (m, 3H), 7.36-7.41 (m, 2H). LC-MS [M+Na]$^+$ *m/z* 419.2 (calcd for $C_{21}H_{21}FN_4O_3$, 396.16).

2.22 *4-Fluoro-N-((*(2*S*,5*R*)*-6-hydroxy-7-oxo-1,6-diazabicyclo[3.2.1]octan-2-yl)(imino)methyl)benzamide (**C5**):* Compound **C5** (109 mg, 55%) was obtained as white solid from **B5** (262 mg, 0.66 mmol) by using the procedure described for **C1** in THF as solvent except ethyl acetate. ^{1}H NMR (400 MHz, DMSO-*d6*): *δ* 1.74-2.09 (m, 4H), 3.07-3.17 (m, 1H), 3.67-3.77 (m, 1H), 4.02-4.11 (m, 1H), 5.41-5.66 (m, 1H), 6.43 (s, 2H), 7.31 (t,*J* = 8.9 Hz, 2H), 7.55 (dd,*J* = 8.7, 5.4 Hz, 2H), 9.20 (s, 1H). ^{19}F NMR (376 MHz, DMSO-*d6*): *δ* -109.6 (s, 1F). LC-MS [M+H]$^+$ *m/z* 307.1 (calcd for $C_{14}H_{15}FN_4O_3$, 306.11).

2.23 (2*S*,5*R*)*-2-(N-(4-Fluorobenzoyl)carbamimidoyl)-7-oxo-1,6-diazabicyclo[3.2.1]octan-6-yl hydrogen sulfate (**A5**):* Compound **A5** (20 mg, 8%) as white solid was prepared from **C5** (200 mg, 0.66 mmol) by using the procedure described for **A1**. ^{1}H NMR (400 MHz, D_2O): *δ* 1.91-2.15 (m, 2H), 2.08-2.20 (m, 2H), 3.39-3.53 (m, 1H), 3.80-3.91 (m, 1H), 3.97-4.09 (m, 1H), 5.68 (s, 1H), 7.17 (t, *J* = 8.9 Hz, 2H), 7.46 (dd, *J* = 8.9, 5.3 Hz, 2H). ^{19}F NMR (376 MHz, D_2O): *δ* -108.9 (s, 1F). LC-MS [M-H]$^-$ *m/z* 385.1 (calcd for $C_{14}H_{15}FN_4O_6S$, 386.07).

2.24 *N-((*(2*S*,5*R*)*-6-(Benzyloxy)-7-oxo-1,6-diazabicyclo[3.2.1]octan-2-yl)(imino)methyl)-4-(trifluoromethyl)benzamide (**B6**):* Compound **B6** (270 mg, 80%) as brown solid was prepared from 4-(trifluoromethyl)benzoic acid (277 mg, 1.46 mmol) and **1** (200 mg, 0.73 mmol) by using the procedure described for **B3**. ^{1}H NMR (400 MHz, DMSO-*d6*): *δ* 1.87-2.09 (m, 4H), 3.06-3.19 (m, 1H), 3.35-3.42 (m, 1H), 4.01-4.11 (m, 1H), 4.78 (s, 2H), 5.65-5.77 (m, 1H), 6.72 (s, 2H), 7.30-7.37 (m, 3H), 7.41-7.48 (m, 2H), 7.67 (d, *J* = 7.9 Hz, 2H), 7.84 (d, *J* = 7.9 Hz, 2H). ^{19}F NMR (376 MHz, DMSO-*d6*)): *δ* -61.1 (s, 3F). LC-MS [M+H]$^+$ *m/z* 447.1 (calcd for $C_{22}H_{21}F_3N_4O_3$, 446.16).

2.25 *N-((*(2*S*,5*R*)*-6-Hydroxy-7-oxo-1,6-diazabicyclo[3.2.1]octan-2-yl)(imino)methyl)-4-(trifluoromethyl)benzamide (**C6**)*: 10% Pd/C (wet, 55% water w/w, 100 mg) was added to a solution of compound **B6** (270 mg, 0.58 mmol) in THF (10 mL). The mixture was stirred under H_2 (balloon) at room temperature overnight, filtered through a pad of celite, and rinsed with EtOAc. The filtrate was concentrated to give a residue, which was purified by silica gel column chromatography eluting with 50% ethyl acetate in petroleum ether to give the title compound **C6** (99 mg, 47%) as a solid. ^{1}H NMR (400 MHz, DMSO-*d6*): *δ* 1.74-1.82 (m, 1H), 1.87-2.09 (m, 2H), 3.06-3.24 (m, 1H), 3.35-3.52 (m, 1H), 4.04-4.13 (m, 1H), 5.74 (brs, 1H), 6.43 (s, 2H), 7.71 (d, *J* = 8.0 Hz, 2H), 7.85 (d, *J* = 8.0 Hz, 2H), 9.20 (s, 1H). ^{19}F NMR (376 MHz, DMSO-*d6*)): *δ* -61.3 (s, 3F). LC-MS [M+H]$^+$ *m/z* 357.1 (calcd for $C_{15}H_{15}F_3N_4O_3$, 356.11).

2.26 *Sodium* (2*S*,5*R*)-*7-Oxo-2-(N-(4-(trifluoromethyl)benzoyl)carbamimidoyl)-1,6-diazabicyclo[3.2.1]octan-6-yl sulfate* ***(A6):*** Compound **A6** (115 mg, 97%) as a white solid was prepared from **C6** (99 mg, 0.27 mmol) by using the procedure described for **A1**. ^{1}H NMR (400 MHz, D_2O): δ 1.94-2.06 (m, 2H), 2.08-2.24 (m, 2H), 3.45-3.56 (m, 1H), 3.71-3.80 (m, 1H), 4.01-4.09 (m, 1H), 5.75 (s, 1H), 7.58(d, *J* = 7.8 Hz, 2H), 7.77 (d, *J* = 7.8 Hz, 2H). ^{19}F NMR (376 MHz, D_2O): δ -62.8 (s, 3F). LC-MS [M-Na]$^-$ *m/z* 435.1 (calcd for $C_{15}H_{14}F_3N_4NaO_6S$, 458.05).

2.27 *N-((*(2*S*,5*R*)*-6-(Benzyloxy)-7-oxo-1,6-diazabicyclo[3.2.1]octan-2-yl)(imino)methyl)-5-(trifluoromethyl)picolinamide* ***(B7):*** Compound **B7** (530 mg, 98%) as a brown solid was prepared from 5-(trifluoromethyl)picolinic acid (345 mg, 1.81 mmol) and **1** (329 mg, 1.20 mmol) by using the procedure described for **B**3. ^{1}H NMR (400 MHz, $CDCl_3$): δ 2.02-2.11 (m, 2H), 2.17-2.35 (m, 2H), 3.50-3.60 (m, 1H), 4.14-4.19 (m, 1H), 4.25-4.36 (m, 1H), 4.80 (d, *J*= 10.4 Hz, 1H), 4.86 (d, *J*= 10.4 Hz, 1H), 5.85 (s, 1H), 7.36-7.39 (m, 3H), 7.40-7.44 (m, 2H), 7.85 (d, *J*=8.2 Hz, 1H), 8.08 (d, *J*=8.2 Hz, 1H), 8.88 (s, 1H). LC-MS [M+Na]$^+$ *m/z* 469.2 (calcd for $C_{22}H_{21}F_3N_4O_3$, 446.16).

2.28 *N-((*(2*S*,5*R*)*-6-Hydroxy-7-oxo-1,6-diazabicyclo[3.2.1]octan-2-yl)(imino)methyl)-5-(trifluoromethyl)picolinamide* ***(C7):*** Compound **C7** (187 mg, 44%) as a white solid was prepared from **B7** (530 mg,1.18 mmol) by using the procedure described for **C6**. ^{1}H NMR(400 MHz, CD_3OD): δ 1.78-1.98 (m, 2H), 2.04-2.16 (m, 2H), 3.33-3.42 (m, 1H), 3.72-3.79 (m, 1H), 4.10-4.20 (m, 1H), 5.77 (s, 1H), 7.79 (d, *J*=8.2 Hz, 1H), 8.19 (d, *J*=8.2 Hz, 1H), 8.86 (s, 1H). LC-MS [M+H]$^+$ *m/z* 357.1 (calcd for $C_{15}H_{15}F_3N_4O_3$, 356.11).

2.29 *Sodium* (2*S*,5*R*)*-7-oxo-2-(N-(5-(trifluoromethyl)picolinoyl)carbamimidoyl)-1,6-diazabicyclo[3.2.1]octan-6-yl sulfate* ***(A7):*** Compound **A7** (223 mg, 98%) as a white solid was prepared from **C7** (187 mg, 0.52 mmol) by using the procedure described for **A1**. ^{1}H NMR (400 MHz, D_2O): δ 1.95-2.05 (m, 2H), 2.09-2.23 (m, 2H), 3.50-3.58 (m, 1H), 3.65-3.71 (m, 1H), 4.02-4.11 (m, 1H), 5.78 (s, 1H), 7.77 (d, *J* = 7.7 Hz, 1H), 8.28 (d, *J* = 7.7 Hz, 1H), 8.69 (s, 1H). ^{19}F NMR (376 MHz, D_2O): δ -62.8 (s, 3F). LC-MS [M-Na]$^-$ *m/z* 435.1 (calcd for $C_{15}H_{14}F_3N_4NaO_6S$, 458.05).

2.30 *N-((*(2*S*,5*R*)*-6-(Benzyloxy)-7-oxo-1,6-diazabicyclo[3.2.1]octan-2-yl)(imino)methyl)-6-(trifluoromethyl)nicotinamide* ***(B8):*** Compound **B8** (364 mg, 75%) as an oil was prepared from 6-(trifluoromethyl)picolinic acid (210 mg, 1.09 mmol) and **1** (200 mg, 0.73 mmol) by using the procedure described for **B2**. ^{1}H NMR (400 MHz, $CDCl_3$): δ 1.90-1.99 (m, 1H), 2.06-2.14 (m, 1H), 2.17-2.31 (m, 2H), 3.31-3.67 (m, 2H), 4.76 (d, *J*= 10.9 Hz, 1H), 4.85 (d, *J*= 10.9 Hz, 1H), 5.68 (s, 1H), 5.76 (s, 1H), 7.32-7.36 (m, 5H), 7.77 (d, *J*=8.0 Hz, 2H), 7.95 (d, *J*=8.0 Hz, 1H), 8.75 (s, 1H). LC-MS [M+Na]$^+$ *m/z* 469.2 (calcd for $C_{22}H_{21}F_3N_4O_3$, 446.16).

2.31 *N-((*(2*S*,5*R*)*-6-Hydroxy-7-oxo-1,6-diazabicyclo[3.2.1]octan-2-yl)(imino)methyl)-6-(trifluoromethyl)nicotinamide* ***(C8):*** Compound **C8** (200 mg, 73%) as a white solid was prepared from **B8** (364 mg, 0.76 mmol) by using the procedure described for **C1**. ^{1}H NMR (400 MHz, CD_3OD): δ 1.69-1.84 (m, 1H), 1.94-2.11 (m, 3H), 3.29-3.40 (m, 1H), 3.47-3.61 (m, 1H), 4.05-4.16 (m, 1H), 5.70 (s, 1H),7.82 (d, *J*=8.1 Hz, 1H), 8.07(d, *J*=8.1 Hz, 1H), 8.74 (s, 1H). LC-MS [M+H]$^+$ *m/z* 357.1 (calcd for $C_{15}H_{15}F_3N_4O_3$, 356.11).

2.32 *Sodium* (2*S*,5*R*)*-7-oxo-2-(N-(6-(trifluoromethyl)nicotinoyl)carbamimidoyl)-1,6-diazabicyclo[3.2.1]octan-6-yl sulfate* ***(A8):*** Compound **A8** (265 mg, 98%) as a white solid was prepared from **C8** (224 mg, 0.62 mmol) by using the procedure described for **A1**. ^{1}H NMR (400 MHz, D_2O): δ 1.88-2.02 (m, 2H), 2.03-2.18 (m, 2H), 3.47-3.56 (m, 1H), 3.63-3.71 (m, 1H), 3.98-4.06 (m, 1H), 5.70 (s, 1H), 7.87 (d, *J* = 8.1 Hz, 1H), 8.08 (dd, *J* = 8.1, 1.7 Hz, 1H), 8.66 (d, *J* = 8.1 Hz, 1H). ^{19}F NMR (376 MHz, DMSO-d_6)): δ -66.6 (s, 3F). LC-MS [M-Na]$^-$ *m/z* 435.1 (calcd for $C_{15}H_{14}F_3N_4NaO_6S$, 458.05).

2.33 *N-(((2S,5R)-6-(Benzyloxy)-7-oxo-1,6-diazabicyclo[3.2.1]octan-2-yl)(imino)methyl)pyrimidine-5-carboxamide* ***(B9):*** Compound **B9** (250 mg, 89%, white solid) was prepared from pyrimidine-5-carboxylic acid (135 mg, 1.09 mmol) and **1** (200 mg, 0.73 mmol) by using the procedure described for **B2**. ^{1}H NMR (400 MHz, DMSO-d_6): δ 1.86-2.09 (m, 4H), 3.22-3.30 (m, 1H), 4.10-4.20 (m, 1H), 4.32-4.39 (m, 1H), 4.79 (s, 2H), 5.70 (brs, 1H), 6.74 (s, 2H), 7.31-7.39 (m, 3H), 7.44-7.50 (m, 2H), 8.94 (s, 2H), 9.32 (s, 1H). LC-MS [M+Na]$^+$ *m/z* 403.2 (calcd for $C_{19}H_{20}N_6O_3$, 380.16).

2.34 *N-(((2S,5R)-6-Hydroxy-7-oxo-1,6-diazabicyclo[3.2.1]octan-2-yl)(imino)methyl)pyrimidine-5-carboxamide* ***(C9):*** Compound **C9** (160 mg, 95%, white solid) was prepared from **B9** (221 mg, 0.58 mmol) by using the procedure described for **C1**. ^{1}H NMR (400 MHz, DMSO-d_6): δ 1.76-1.83 (m, 1H), 1.90-2.09 (m, 3H), 3.11-3.18 (m, 1H), 3.38-3.61 (m, 1H), 4.06-4.18 (m, 1H), 5.62-5.81 (m, 1H), 6.44 (s, 2H), 8.96 (s, 2H), 9.26 (s, 1H), 9.30 (s, 1H). LC-MS [M+Na]$^+$ *m/z* 313.1 (calcd for $C_{12}H_{14}N_6O_3$, 290.11).

2.35 *Sodium (2S,5R)-7-oxo-2-(N-(pyrimidine-5-carbonyl)carbamimidoyl)-1,6-diazabicyclo[3.2.1]octan-6-yl sulfate* ***(A9):*** Compound **A9** (32 mg, 15%) as a white solid was prepared from **C9** (160 mg, 0.55 mmol) and SO_3-NMe_3 (200 mg, 1.43 mmol) by using the procedure described for **A1**. ^{1}H NMR (400 MHz, DMSO-d_6): δ 1.92-2.09 (m, 2H), 2.10-2.25 (m, 2H), 3.55-3.66 (m, 1H), 3.76-3.85 (m, 1H), 4.05-4.14 (m, 1H), 5.73 (s, 1H), 8.89 (s, 2H), 9.195 (s, 1H). LC-MS [M-Na]$^-$ *m/z* 369.0 (calcd for $C_{12}H_{13}N_6NaO_6S$, 392.05).

2.36 *N-(((2S,5R)-6-(Benzyloxy)-7-oxo-1,6-diazabicyclo[3.2.1]octan-2-yl)(imino)methyl)pyrimidine-4-carboxamide* ***(B10):*** Compound **B10** (222 mg, 80%) as a yellow oil was prepared from pyrimidine-4-carboxylic acid (181 mg, 1.46 mmol) and **1** (200 mg, 0.73 mmol) by using the procedure described for **B3**. LC-MS [M+H]$^+$ *m/z* 381.2 (calcd for $C_{19}H_{20}N_6O_4$, 380.16).

2.37 *N-(((2S,5R)-6-hydroxy-7-oxo-1,6-diazabicyclo[3.2.1]octan-2-yl)(imino)methyl)pyrimidine-4-carboxamide* ***(C10):*** 10% Pd/C (wet, 55% water w/w, 100 mg) was added to a solution of compound **B10** (222 mg, 0.58 mmol)in MeOH (10 mL). The mixture was stirred under H_2 (balloon) at room temperature overnight, filtered through a pad of celite, and rinsed with EtOAc. The filtrate was concentrated to give a residue, which was purified by silica gel column chromatography eluting with 50% ethyl acetate in petroleum ether to give the title compound **C10** (60 mg, 36%) as a solid.^{1}H NMR (400 MHz, DMSO-d_6): δ 1.87-1.99 (m, 3H), 2.08-2.14 (m, 1H), 3.23-3.31 (m, 1H), 3.31-3.51 (m, 1H), 3.98-4.09 (m, 1H), 5.81 (s, 1H), 7.79 (t, *J* = 5.0 Hz, 1H), 9.03 (d, *J* = 5.0 Hz, 1H), 9.19 (s, 1H), 9.29 (d, *J* = 3.9 Hz, 1H), 9.33 (s, 1H). LC-MS [M+H]$^+$ *m/z* 291.2 (calcd for $C_{12}H_{14}N_6O_3$, 290.11).

2.38 *Sodium (2S,5R)-7-oxo-2-(N-(pyrimidine-4-carbonyl)carbamimidoyl)-1,6-diazabicyclo[3.2.1]octan-6-yl sulfate* ***(A10):*** Compound **A10** (80 mg, 97%) as white solid was prepared from **C10** (60 mg, 0.21 mmol) and $SO_3{\cdot}NMe_3$ (74 mg, 0.53 mmol) by using the procedure described for **A1**. ^{1}H NMR (400 MHz, D_2O): δ 1.93-2.06 (m, 2H), 2.09-2.25 (m, 2H), 3.52-3.61 (m, 1H), 3.70-3.76 (m, 1H), 4.02-4.13 (m, 1H), 5.77 (s, 1H), 7.70 (d, *J* = 5.3 Hz, 1H), 8.93 (d, *J* = 5.3 Hz, 1H), 9.17 (s, 1H). LC-MS [M-Na]$^-$ *m/z* 369.0 (calcd for $C_{12}H_{13}N_6NaO_6S$, 392.05).

2.39 *N-(((2S,5R)-6-(Benzyloxy)-7-oxo-1,6-diazabicyclo[3.2.1]octan-2-yl)(imino)methyl)-2-(pyrimidin-2-yl)acetamide* ***(B11):*** Compound **B11** (621 mg, 43%) as a yellow solid was prepared from 2-(pyrimidin-2-yl)acetic acid (0.76 g, 5.74 mmol) and **1** (1.0g, 3.65 mmol) by using the procedure described for **B3**. LC-MS [M+H]$^+$ *m/z* 395.1 (calcd for $C_{19}H_{20}N_6O_3$, 394.18).

2.40 *N-(((2S,5R)-6-Hydroxy-7-oxo-1,6-diazabicyclo[3.2.1]octan-2-yl)(imino)methyl)-2-(pyrimidin-2-yl)acetamide* ***(C11):*** Compound **C11** (126 mg, 33%) as a white solid was prepared from **B11** (500 mg, 1.27 mmol) by using the procedure described for **C10**. ^{1}H NMR (400 MHz, DMSO-d_6): δ 1.68-1.78 (m, 1H), 1.84-2.05 (m, 3H), 3.20-3.26 (m, 1H), 3.77-3.96 (m, 2H), 4.06-4.12 (m, 2H), 5.70 (s, 1H), 6.40 (s, 2H), 7.39 (t, *J* = 4.3 Hz, 1H), 8.75 (d, *J* = 4.7 Hz, 2H), 9.28 (s, 1H). LC-MS [M+H]$^+$ *m/z* 305.1 (calcd for $C_{13}H_{16}N_6O_3$, 304.13).

2.41 *Sodium* (2*S*,5*R*)*-7-oxo-2-(N-(2-(pyrimidin-2-yl)acetyl)carbamimidoyl)-1,6-diazabicyclo[3.2.1]octan-6-yl sulfate* ***(A11)****:* Compound **A11** (158 mg, 94%) as a white solid was prepared from **C11** (126 mg，0.41 mmol) and SO_3-NMe_3 (138 mg, 1.01 mmol) by using the procedure described for **A1**. ^{1}H NMR (400 MHz, D_2O): δ 1.80-1.92 (m, 1H), 1.94-2.05 (m, 1H), 2.08-2.20 (m, 2H), 3.56 (t, *J* = 12.8 Hz, 1H), 3.83-3.92 (m, 1H), 3.95-4.02 (m, 1H), 4.13-4.28 (m, 2H), 5.75 (d, *J* = 4.7 Hz, 1H), 7.46 (t, *J* = 5.1 Hz, 1H), 8.73 (d, *J* = 5.1 Hz, 2H). LC-MS $[M-Na]^-$ *m/z* 383.1 (calcd for $C_{13}H_{15}N_6NaO_6S$, 406.07).

2.42 *N-((*(2*S*,5*R*)*-6-(Benzyloxy)-7-oxo-1,6-diazabicyclo[3.2.1]octan-2-yl)(imino)methyl)-3-(pyridin-2-yl)propanamide* ***(B12)****:* Compound **B12** (111 mg, 78%) as a white solid was prepared from 3-(pyridin-2-yl)propanoic acid (82.7 mg, 0.54 mmol) and **1** (98 mg, 0.36 mmol) by using the procedure described for **B2**. LC-MS $[M+H]^+$ *m/z* 408.2 (calcd for $C_{22}H_{25}N_5O_3$, 407.20).

2.43 *Sodium*(2*S*,5*R*)*-7-oxo-2-(N-(3-(pyridin-2-yl)propanoyl)carbamimidoyl)-1,6-diazabicyclo[3.2.1]octan-6-yl sulfate(****A12****):* A mixture of compound **B12** (111 mg, 0.27 mmol), SO_3·NMe_3 (57 mg, 0.41 mmol), TEA (82 mg, 0.81 mmol) and 10% Pd/C (wet, 55% water w/w, 50 mg) in MeOH/water (2/2 mL) was stirred under H_2 (balloon) at room temperature overnight. The reaction was filtered through a pad of celite, and rinsed with EtOAc. The filtrate was concentrated to give a residue, which was purified by Dowex-50wx Na^+ resin using water and lyophilized to give **A12** (30 mg, 28%) as a white solid.^{1}H NMR (400 MHz, D_2O): δ 1.48-1.59 (m, 1H), 1.74-1.82 (m, 1H), 1.85-1.97 (m, 2H), 2.73-2.81 (m, 2H), 2.91-2.98 (m, 2H), 3.06-3.16 (m, 1H), 3.67-3.80 (m, 2H), 5.45-5.48 (m, 1H), 7.20-7.27 (m, 2H), 7.70-7.76 (m, 1H), 8.27 (d, *J* = 4.7 Hz, 1H). LC-MS $[M-Na]^-$ *m/z* 396.1 (calcd for $C_{15}H_{18}N_5NaO_6S$, 419.09).

2.44 *N-((*(2*S*,5*R*)*-6-(Benzyloxy)-7-oxo-1,6-diazabicyclo[3.2.1]octan-2-yl)(imino)methyl)furan-2-carboxamide (****B13****):* Compound **B13** (202mg, 75%, as an oil) was prepared from furan-2-carboxylic acid (122 mg, 1.09 mmol) and **1** (200 mg, 0.73 mmol) by using the procedure described for **B2**. ^{1}H NMR (400 MHz, $CDCl_3$): δ 1.88-1.98 (m, 1H), 2.03-2.11 (m, 1H), 2.14-2.20 (m, 1H), 2.24-2.35 (m, 1H), 3.45-3.54 (m, 1H), 4.20-4.29 (m, 1H), 4.60 (d, *J* = 13.3 Hz, 1H), 4.87 (d, *J* = 10.4 Hz, 1H), 4.92 (d, *J* = 10.4 Hz, 1H), 5.36 (brs, 2H), 5.73 (d, *J* = 4.8 Hz, 1H), 6.51 (dd, *J* = 3.6, 1.7 Hz, 1H), 7.15 (d, *J* = 3.6 Hz, 1H), 7.54 (d, *J* = 1.7 Hz, 1H). LC-MS $[M+Na]^+$ *m/z* 391.2 (calcd for $C_{19}H_{20}N_4O_4$, 368.15).

2.45 *N-((*(2*S*,5*R*)*-6-Hydroxy-7-oxo-1,6-diazabicyclo[3.2.1]octan-2-yl)(imino)methyl)furan-2-carboxamide (****C13****):* Compound **C13** (200 mg, 66%) as a white solid was prepared from **B13** (359 mg, 1 mmol) by using the procedure described for **C1**. ^{1}H NMR(400 MHz, CD_3OD): δ 1.89-1.91 (m, 2H), 2.01-2.14 (m, 2H), 3.24-3.41 (m, 1H), 4.03-4.13 (m, 1H), 4.33-4.41 (m, 1H), 5.53-5.63 (m, 1H), 6.48-6.51 (m, 1H), 7.05-7.08 (m, 1H), 7.60-7.64 (m,1H). LC-MS $[M+Na]^+$ *m/z* 301.1 (calcd for $C_{12}H_{14}N_4O_4$, 278.10).

2.46 (2*S*,5*R*)*-2-(N-(Furan-2-carbonyl)carbamimidoyl)-7-oxo-1,6-diazabicyclo[3.2.1]octan-6-yl hydrogen sulfate* ***(A13)****:* Compound **A13** (25 mg, 11% in two steps) as white solid was prepared from **C13** (200 mg, 0.66 mmol) and SO_3-NMe_3 (193 mg, 1.38 mmol) by using the procedure described for **A1**. ^{1}H NMR (400 MHz, D_2O): ^{1}H NMR (400 MHz, D_2O): δ 1.81-1.90 (m, 1H), 1.92-2.00 (m, 1H), 2.04-2.12 (m, 2H), 3.39-3.50 (m, 1H), 3.98-4.06 (m, 1H), 4.49 (d, *J* = 13.9 Hz, 1H), 5.60 (s, 1H), 6.50 (dd, *J* = 3.6, 1.7 Hz, 1H), 7.05 (d, *J* = 3.6 Hz, 1H), 7.58 (d, *J* = 1.7 Hz, 1H). LC-MS $[M-H]^-$ *m/z* 357.0 (calcd for $C_{12}H_{14}N_4O_7S$, 358.06).

2.47 *N-((*(2*S*,5*R*)*-6-(Benzyloxy)-7-oxo-1,6-diazabicyclo[3.2.1]octan-2-yl)(imino)methyl)oxazole-5-carboxamide* ***(B14)****:* Compound **B14** (204 mg, 75%) as a white solid was prepared from oxazole-5-carboxylic acid (123 mg, 1.09 mmol) and **1** (200 mg, 0.73 mmol) by using the procedure described for **B2**. LC-MS $[M+Na]^+$ *m/z* 392.3 (calcd for $C_{18}H_{19}N_5O_4$, 369.14).

2.48 *N-((*(2*S*,5*R*)*-6-Hydroxy-7-oxo-1,6-diazabicyclo[3.2.1]octan-2-yl)(imino)methyl)oxazole-5-carboxamide (****C14****):* Compound **C14** (160 mg, crude) as a white solid was prepared from **B14** (204 mg, 0.55 mmol) by using the procedure described for **C10**. ^{1}H NMR (400 MHz, CD_3OD): δ 1.71-2.01 (m, 4H), 3.43-3.49 (m, 1H), 4.17-4.23 (m, 1H), 4.32-4.43 (m, 1H), 5.74 (s, 1H), 7.67 (s, 1H), 7.84 (s, 1H), 8.41 (s, 1H). LC-MS $[M+H]^+$ *m/z* 280.1 (calcd for $C_{11}H_{13}N_5O_4$, 379.10).

2.49 *Sodium* (2*S*,5*R*)*-2-(N-(oxazole-5-carbonyl)carbamimidoyl)-7-oxo-1,6-diazabicyclo[3.2.1]octan-6-yl sulfate* ***(A14):*** Compound **A14** (20 mg, 10% in two steps) as a white solid was prepared from **C14** (160 mg obtained above) and SO_3·NMe_3 (300 mg, 2.13 mmol) by using the procedure described for **A1**. ^{1}H NMR (400 MHz, D_2O): δ 1.89-2.08 (m, 2H), 2.12-2.14 (m, 2H), 3.51-3.65 (m, 1H), 4.08-4.18 (m, 1H), 4.43-4.51 (m, 1H), 5.68 (d, *J* = 4.6 Hz, 1H), 7.74 (s, 1H), 8.29 (s, 1H). LC-MS $[M-Na]^-$ *m/z* 358.1 (calcd for $C_{11}H_{12}N_5NaO_7S$, 381.04).

2.50 *N-((*(2*S*,5*R*)*-6-(Benzyloxy)-7-oxo-1,6-diazabicyclo[3.2.1]octan-2-yl)(imino)methyl)oxazole-4-carboxamide* ***(B15):*** Compound **B15** (202 mg, 73%) as a white solid was prepared from oxazole-4-carboxylic acid (123 mg, 1.09 mmol) and **1** (200 mg, 0.73 mmol) by using the procedure described for **B2**. LC-MS $[M+Na]^+$ *m/z* 392.1(calcd for $C_{18}H_{19}N_5O_4$, 369.14).

2.51 *N-((*(2*S*,5*R*)*-6-Hydroxy-7-oxo-1,6-diazabicyclo[3.2.1]octan-2-yl)(imino)methyl)oxazole-4-carboxamide* ***(C15):*** Compound **C15** (203 mg, 72%) as a white solid was prepared from **B15** (269 mg, 0.73 mmol) by using the procedure described for **C1**. LC-MS $[M+H]^+$ *m/z* 280.1 (calcd for $C_{11}H_{13}N_5O_4$, 279.10).

2.52 *Sodium* (2*S*,5*R*)*-2-(N-(oxazole-4-carbonyl)carbamimidoyl)-7-oxo-1,6-diazabicyclo[3.2.1]octan-6-yl sulfate* ***(A15):*** Compound **A15** (20 mg, 8%) as a white solid was prepared from **C15** (203 mg, 0.72 mmol) and SO_3·NMe_3 (200 mg, 1.42 mmol) by using the procedure described for **A1**. ^{1}H NMR (400 MHz, D_2O): δ 1.83-2.02 (m, 2H), 2.07-2.18 (m, 2H), 3.49-3.56 (m, 1H), 3.99-4.09 (m, 1H), 4.40-4.49 (m, 1H), 5.72 (brs, 1H), 8.16 (s, 1H), 8.30 (s, 1H). LC-MS $[M-Na]^-$ *m/z* 358.1 (calcd for $C_{11}H_{12}N_5NaO_7S$, 381.04).

2.53 *N-((*(2*S*,5*R*)*-6-(Benzyloxy)-7-oxo-1,6-diazabicyclo[3.2.1]octan-2-yl)(imino)methyl)cyclohexanecarboxamide* ***(B16):*** Compound **B16** (960 mg, 85%) as a white solid was prepared from cyclohexanecarboxylic acid (561 mg, 4.38 mmol)and **1** (800 mg, 2.92 mmol) by using the procedure described for **B3**. LC-MS $[M+H]^+$ *m/z* 385.2 (calcd for $C_{21}H_{28}N_4O_3$, 384.22).

2.54 *N-((*(2*S*,5*R*)*-6-Hydroxy-7-oxo-1,6-diazabicyclo[3.2.1]octan-2-yl)(imino)methyl)cyclohexanecarboxamide* ***(C16):*** Compound **C16** (137 mg, 22%) as a white solid was prepared from **B16** (800 mg, 2.08 mmol) by using the procedure described for **C6** in THF as solvent. ^{1}H NMR (400 MHz, DMSO-d_6): δ 1.10-1.19 (m, 1H), 1.23-1.35 (m, 4H), 1.58-1.76 (m, 7H), 1.83-1.94 (m, 1H), 1.97-2.04 (m, 1H), 2.53-2.60 (m, 1H), 3.18 (t, *J* = 11.4 Hz, 1H), 3.76-3.86 (m, 1H), 3.87-3.97 (m, 1H), 5.66 (s, 1H), 6.48 (s, 2H), 9.28 (s, 1H). LC-MS $[M+H]^+$ *m/z* 295.1 (calcd for $C_{14}H_{22}N_4O_3$, 294.17).

2.55 *Sodium* (2*S*,5*R*)*-2-(N-(cyclohexanecarbonyl)carbamimidoyl)-7-oxo-1,6-diazabicyclo[3.2.1]octan-6-yl sulfate* ***(A16):*** Compound **A16** (170mg, 99%) as a white solid was prepared from **C16** (124 mg, 0.42 mmol) and SO_3·NMe_3 (120 mg, 0.86 mmol) by using the procedure described for **A1**. ^{1}H NMR (400 MHz, D_2O): δ 1.06-1.29 (m, 5H), 1.55-1.76 (m, 6H), 1.93-2.00 (m, 1H), 2.04-2.15 (m, 2H), 2.59-2.68 (m, 1H), 3.50 (t, *J* = 12.4 Hz, 1H), 3.90-3.97 (m, 1H), 4.13 (d, *J* = 13.1 Hz, 1H), 5.69 (d, *J* = 4.6 Hz, 1H). LC-MS $[M-Na]^-$ *m/z* 373.1 (calcd for $C_{14}H_{21}NaN_4O_6S$, 396.11).

2.56 *4-Acetamido-N-((*(2*S*,5*R*)*-6-(benzyloxy)-7-oxo-1,6-diazabicyclo[3.2.1]octan-2-yl)(imino)methyl)cyclohexane-1-carboxamide* ***(B17):*** Compound **B17** (180 mg, 55%, as an oil) was prepared from 4-acetamidocyclohexane-1-carboxylic acid (150 mg, 0.81 mmol)and **1** (200 mg, 0.73 mmol) by using the procedure described for **B2**. ^{1}H NMR (400 MHz, $CDCl_3$): δ 1.54-1.86 (m, 8H), 1.94 (s, 3H), 2.04-2.12 (m, 4H), 2.31-2.38 (m, 1H), 3.32-3.42 (m, 1H), 3.69-3.83 (m, 2H), 3.99-4.08 (m, 1H), 4.84 (d, *J* = 10.7 Hz, 1H), 4.93 (d, *J* = 10.7 Hz, 1H), 5.29-5.39 (m, 2H), 5.79 (s, 1H), 7.37-7.44 (m, 5H). LC-MS $[M+H]^+$ *m/z* 442.2 (calcd for $C_{23}H_{31}N_5O_4$, 441.24).

2.57 *4-Acetamido-N-((*(2*S*,5*R*)*-6-hydroxy-7-oxo-1,6-diazabicyclo[3.2.1]octan-2-yl)(imino)methyl)cyclohexane-1-carboxamide* ***(C17):*** Compound **C17** (150 mg) as a white solid was prepared from **B17** (180 mg, 0.41 mmol) in THF by using the procedure described for **C6**. The white solid obtained was directly used for next step without further purification. LC-MS $[M+H]^+$ *m/z* 352.2 (calcd for $C_{16}H_{25}N_5O_4$, 351.19).

***2.58** Sodium (2S,5R)-2-(N-(4-acetamidocyclohexane-1-carbonyl)carbamimidoyl)-7-oxo-1,6-diazabicyclo[3.2.1]octan-6-yl sulfate (**A17**):* Compound **A17** (150mg, 85% in two steps) as a white solid was prepared from **C17** (150 mg obtained above) and SO_3-NMe_3 (100 mg, 0.72 mmol) by using the procedure described for **A1**. ^1H NMR (400 MHz, D_2O): δ 1.17-1.29 (m, 2H), 1.32-1.45 (m, 2H), 1.65-1.80 (m, 3H), 1.81-1.98 (m, 6H), 2.03-2.17 (m, 2H), 2.57-2.67 (m, 1H), 3.44-3.55 (m, 2H), 3.90-4.01 (m, 1H), 4.12 (d, *J* = 13.7 Hz, 1H), 5.68 (d, *J* = 4.3 Hz, 1H). LC-MS $[M-Na]^-$ *m/z* 430.1 (calcd for $C_{16}H_{24}N_5NaO_7S$, 453.13).

***2.59** tert-Butyl 4-(((2S,5R)-6-(benzyloxy)-7-oxo-1,6-diazabicyclo[3.2.1]octan-2-yl)(imino)methyl)carbamoyl)piperidine-1-carboxylate (**B18**):* Compound **B18** (230 mg, 65%) as an oil was prepared from 1-(*tert*-butoxycarbonyl)piperidine-4-carboxylic acid (250 mg, 1.09 mmol)and **1** (200 mg, 0.73 mmol) by using the procedure described for **B2**. LC-MS $[M+Na]^+$ *m/z* 508.3 (calcd for $C_{25}H_{35}N_5O_5$, 485.26).

***2.60** tert-Butyl 4-(((2S,5R)-6-hydroxy-7-oxo-1,6-diazabicyclo[3.2.1]octan-2-yl)(imino)methyl)carbamoyl)piperidine-1-carboxylate (**C18**):* Compound **C18** (180 mg, 96%) as a white solid was prepared from **B18** (230 mg, 0.47 mmol) in MeOH by using the procedure described for **C10**. The white solid obtained was directly used for next step without further purification. LC-MS $[M+Na]^+$ *m/z* 418.2 (calcd for $C_{18}H_{29}N_5O_5$, 395.22).

***2.61** Sodium (2S,5R)-7-oxo-2-(N-(piperidine-4-carbonyl)carbamimidoyl)-1,6-diazabicyclo[3.2.1]octan-6-yl sulfate (**A18**):* A mixture of compound **C18** (180 mg, 0.45 mmol), $SO_3\cdot NMe_3$ (300 mg, 2.15 mmol) and TEA (2 mL, 14.45 mmol)in THF/water (10/10 mL) was stirred at room temperature overnight. The reaction mixture was concentrated under reduced pressure to provide a residue, which was purified by preparative HPLC on an Agilent 10 prep-C18 250 × 21.2 mm column and lyophilized, followed byDowex-50wx Na^+ resin using water as an eluting solvent to give the desired intermediate (55 mg, 25% in two steps, sodium salt) as a white solid. ^1H NMR (400 MHz, D_2O): δ 1.33 (s, 9H), 1.35-1.47 (m, 2H), 1.62-1.76 (m, 3H), 1.90-1.98 (m, 1H), 2.02-2.13 (m, 2H), 2.72-2.83 (m, 2H), 2.84-2.92 (m, 1H), 3.51 (t, *J* = 12.1 Hz, 1H), 3.90-4.01 (m, 3H), 4.09-4.15 (m, 1H), 5.66 (d, *J* = 4.4 Hz, 1H). LC-MS $[M-Na]^-$*m/z* 474.1 ($C_{18}H_{28}N_5NaO_8S$, 497.16). In the next step, TFA (1.8 mL) was added to a suspension of the intermediate obtained above (350 mg, 0.63 mmol) in CH_2Cl_2 (6 mL) at 0 °C. The reaction mixture was stirred for 3.5 hours at 0 °C, and then concentrated under reduced pressure to dryness to give a residue. The residue was dissolved with CH_2Cl_2 (15 mL) and extracted with water (2 × 10 mL). The aqueous layer was freeze-dried and purified by Dowex-50wx Na^+ resin, using water as an elution solvent to give **A18** (15 mg, 6%) as a white powder. ^1H NMR (400 MHz, D_2O): δ 1.62-1.74 (m, 3H), 1.80-1.92 (m, 3H), 1.94-2.07 (m, 2H), 2.85-3.05 (m, 3H), 3.31 (d, *J* = 12.6 Hz, 2H), 4.47 (t, *J* = 12.6 Hz, 1H), 3.83-3.91 (m, 1H), 4.02 (d, *J* = 12.6 Hz, 1H), 5.59 (d, *J* = 4.0 Hz, 1H). LC-MS $[M-Na]^-$ *m/z* 374.1 (calcd for $C_{13}H_{20}NaN_5O_6S$, 397.10).

***2.62** (3R)-1-Acetyl-N-(((2S,5R)-6-(benzyloxy)-7-oxo-1,6-diazabicyclo[3.2.1]octan-2-yl)(imino)methyl)piperidine-3-carboxamide (**B19**):* Compound **B19** (320 mg, 93%) as a pale brown solid was prepared from (*R*)-1-acetylpiperidine-3-carboxylic acid (**3**) (215 mg, 1.26 mmol) and **1** (220 mg, 0.80 mmol) by using the procedure described for **B3**. ^1H NMR (400 MHz, DMSO-d_6): δ 1.39-1.54 (m, 2H), 1.56-1.74 (m, 2H), 1.80-1.93 (m, 2H), 1.93-2.05 (m, 5H), 2.51-2.62 (m, 1H), 2.71-2.88 (m, 1H), 2.94-3.28 (m, 2H), 3.68-3.95 (m, 3H), 4.17-4.38 (m, 1H), 4.78-4.92 (m, 2H), 5.57-5.70 (m, 1H), 6.65-6.86 (m, 2H), 7.32-7.42 (m, 3H), 7.46-7.55 (m, 2H). LC-MS $[M+H]^+$ *m/z* 428.3 (calcd for $C_{22}H_{29}N_5O_4$, 427.22).

***2.63** (3R)-1-Acetyl-N-(((2S,5R)-6-hydroxy-7-oxo-1,6-diazabicyclo[3.2.1]octan-2-yl)(imino)methyl)piperidine-3-carboxamide (**C19**):* Compound **C19** (170 mg, 67%) as a white solid was prepared from **B19** (320 mg, 0.75 mmol) in THF as solvent by using the procedure described for **C6**. ^1H NMR (400 MHz, DMSO-d_6): δ 1.38-1.54 (m, 2H), 1.56-1.80 (m, 3H), 1.83-2.05 (m, 6H), 2.52-2.63 (m, 1H), 2.65-2.86 (m, 1H), 2.93-3.24 (m, 2H), 3.67-3.96 (m, 3H), 4.24-4.36 (m, 1H), 5.58-5.70 (m, 1H), 6.40-6.54 (m, 2H), 9.31 (s, 1H). LC-MS $[M+H]^+$ *m/z* 338.2 (calcd for $C_{15}H_{23}N_5O_4$, 337.18).

2.64 *Sodium* (2*S*,5*R*)-*2-(N-((R)-1-acetylpiperidine-3-carbonyl)carbamimidoyl)-7-oxo-1,6-diazabicyclo[3.2.1]octan-6-yl sulfate* ***(A19):*** Compound **A19** (215 mg, 98%) as a white solid was prepared from **C19** (170mg， 0.50 mmol) and SO_3·NMe_3 (139 mg, 1.0 mmol) by using the procedure described for **A1**. ^{1}H NMR (400 MHz, D_2O): δ 1.47-1.66 (m, 2H), 1.68-1.87 (m, 2H), 1.96-2.06 (m, 1H), 2.09 (s, 3H), 2.11-2.21 (m, 2H), 2.81-3.03 (m, 2H), 3.11-3.19 (m, 0.5H), 3.30-3.38 (m, 0.5H), 3.55-3.64 (m, 1H), 3.74-3.84 (m, 1H), 3.96-4.14 (m, 2.5H), 4.20-4.26 (m, 0.5H), 5.96-5.74 (m, 1H). LC-MS [M-Na]$^-$ *m/z* 416.2. (calcd for $C_{15}H_{22}NaN_5O_7S$, 439.11).

2.65 *(3S)-1-Acetyl-N-((*(2*S*,5*R*)*-6-(benzyloxy)-7-oxo-1,6-diazabicyclo[3.2.1]octan-2-yl)(imino)methyl)piperidine-3-carboxamide* ***(B20):*** Compound **B20** (278 mg, 81%) as a pale yellow solid was prepared from (*S*)-1-acetylpiperidine-3-carboxylic acid (**4)** (215 mg, 1.26 mmol) and **1** (220 mg, 0.80 mmol) by using the procedure described for **B3**. ^{1}H NMR (400 MHz, DMSO-d_6): δ 1.38-1.55 (m, 2H), 1.57-1.77 (m, 3H), 1.79-1.99 (m, 3H), 2.01 (s, 3H), 2.50-2.61 (m, 1H), 2.71-2.89 (m, 1H), 2.96-3.26 (m, 2H), 3.67-4.03 (m, 3H), 4.16-4.32 (m, 1H), 4.74-4.92 (m, 2H), 5.57-5.71 (m, 1H), 6.64-6.86 (m, 2H), 7.31-7.42 (m, 3H), 7.44-7.56 (m, 2H). LC-MS [M+H]$^+$ *m/z* 428.3 (calcd for $C_{22}H_{29}N_5O_4$, 427.22).

2.66 *(3S)-1-Acetyl-N-((*(2*S*,5*R*)*-6-hydroxy-7-oxo-1,6-diazabicyclo[3.2.1]octan-2-yl)(imino)methyl)piperidine-3-carboxamide* ***(C20):*** Compound **C20** (136 mg, 63%) as a white solid was prepared from **B20** (276 mg, 0.64 mmol) in THF as solvent by using the procedure described for **C6**. ^{1}H NMR (400 MHz, DMSO-d_6): δ 1.43-1.55 (m, 2H), 1.57-1.65 (m, 1H), 1.67-1.93 (m, 4H), 1.94-2.02 (m, 1H), 2.02 (s, 3H), 2.57-2.73 (m, 1H), 2.80-2.90 (m, 1H), 2.99-3.23 (m, 2H), 3.66-3.90 (m, 2H), 4.00-4.12 (m, 1H), 4.18-4.32 (m, 1H), 5.61-5.70 (m, 1H), 6.42-6.48 (m, 2H), 9.26 (s, 1H). LC-MS [M+H]$^+$ *m/z* 338.2 (calcd for $C_{15}H_{23}N_5O_4$, 337.18).

2.67 *Sodium* (2*S*,5*R*)-*2-(N-((S)-1-acetylpiperidine-3-carbonyl)carbamimidoyl)-7-oxo-1,6-diazabicyclo[3.2.1]octan-6-yl sulfate* ***(A20):*** Compound **A20** (160 mg, 97%) as a white solid was prepared from **C20** (133 mg， 0.395 mmol) and SO_3·NMe_3 (139 mg, 1.0 mmol) by using the procedure described for **A1**. ^{1}H NMR (400 MHz, D_2O): δ 1.45-1.64 (m, 2H), 1.75-1.87 (m, 2H), 1.91-2.03 (m, 1H), 2.08 (s, 1.5H), 2.09 (s, 1.5H), 2.09-2.21 (m, 2H), 2.81-3.10 (m, 3H), 3.29-3.39 (m, 1H), 3.53-3.64 (m, 1H), 3.72-3.84 (m, 1H), 4.04-4.15 (m, 2H), 5.68-5.73 (m, 1H). LC-MS [M-Na]$^-$ *m/z* 416.2. (calcd for $C_{15}H_{22}NaN_5O_7S$, 439.11).

2.68 *N-((*(2*S*,5*R*)*-6-(Benzyloxy)-7-oxo-1,6-diazabicyclo[3.2.1]octan-2-yl)(imino)methyl)-1-methylpiperidine-4-carboxamide* ***(B21):*** Compound **B21** (200 mg, 68%) as a white solid was prepared from 1-methylpiperidine-4-carboxylic acid (160 mg, 1.09 mmol) and **1** (220 mg, 0.80 mmol) by using the procedure described for **B2**. LC-MS [M+H]$^+$ *m/z* 400.1 (calcd for $C_{21}H_{29}N_5O_3$, 399.23).

2.69 *N-((*(2*S*,5*R*)*-6-Hydroxy-7-oxo-1,6-diazabicyclo[3.2.1]octan-2-yl)(imino)methyl)-1-methylpiperidine-4-carboxamide* ***(C21):*** Compound **C21** (100 mg) as a white solid was prepared from **B21** (200 mg, 0.50 mmol) in THF as solvent by using the procedure described for **C6**. LC-MS [M+H]$^+$ *m/z* 310.2 (calcd for $C_{14}H_{23}N_5O_3$, 309.18).

2.70 *Sodium* (2*S*,5*R*)-*2-(N-(1-methylpiperidine-4-carbonyl)carbamimidoyl)-7-oxo-1,6-diazabicyclo[3.2.1]octan-6-yl sulfate* ***(A21):*** Compound **A21** (20 mg, 10% in two steps) as a white solid was prepared from **C21** (100 mg obtained above) and SO_3·NMe_3 (100 mg, 0.72 mmol) by using the procedure described for **A1**. ^{1}H NMR (400 MHz, D_2O): δ 1.73-1.86 (m, 3H), 1.92-2.04 (m, 3H), 2.05-2.16 (m, 2H), 2.78 (s, 3H), 2.95-3.07 (m, 3H), 3.47-3.61 (m, 3H), 3.97 (d, *J* = 11.17 Hz, 1H), 4.11 (d, *J* = 14.1 Hz, 1H), 5.68 (s, 1H). LC-MS [M-Na]$^-$ *m/z* 388.1 (calcd for $C_{14}H_{22}NaN_5O_6S$, 411.12).

2.71 *(2R)-1-Acetyl-N-((*(2*S*,5*R*)*-6-((tert-butyldimethylsilyl)oxy)-7-oxo-1,6-diazabicyclo[3.2.1]octan-2-yl)(imino)methyl)piperidine-2-carboxamide* ***(B22):*** Compound **B22** (292 mg, 62%) as a white solid was prepared from (*R*)-1-acetylpiperidine-2-carboxylic acid (**5**, 380 mg, 1.94 mmol) and **2** (400 mg, 1.34 mmol) by using the procedure described for **B2**. ^{1}H NMR (400 MHz, $CDCl_3$): δ 0.17 (s, 6H), 0.92 (s, 9H), 1.21-1.43 (m, 2H), 1.59-1.73 (m, 4H), 1.87-1.90 (m, 3H), 2.11 (s, 3H), 3.18-3.25 (m, 1H), 3.40-3.47 (m, 1H), 3.62-3.65 (m, 2H), 3.75-3.87 (m, 2H), 5.32 (s, 1H), 5.41-5.44 (m, 2H), 5.63 (s, 1H). LC-MS [M+Na]$^+$ *m/z* 474.3 (calcd for $C_{21}H_{37}N_5O_4Si$, 451.26).

2.72 *(2R)-1-Acetyl-N-(((2S,5R)-6-hydroxy-7-oxo-1,6-diazabicyclo[3.2.1]octan-2-yl)(imino)methyl)piperidine-2-carboxamide* (**C22**): TBAF (1M solution in THF, 0.7 mL, 0.7 mmol) was added to a solution of Compound **B22** (292 mg, 0.64 mmol) in THF (5 mL) dropwise at 0 °C. The reaction mixture was stirred at this temperature for 3 hours. The solvent was removed to obtain a thick slurry, which was subjected to silica gel column chromatography eluting with 6–8% MeOH in CH_2Cl_2, and subsequent recrystallization from EtOAc and petroleum ether to afford the title compound **C22** (187 mg, 92%) as a white solid. ^{1}H NMR (400 MHz, DMSO-d_6): δ 1.59-1.74 (m, 4H), 1.82-1.93 (m, 2H), 1.98-2.03 (m, 2H), 2.04 (s, 3H), 3.2 (t, J = 12.3 Hz, 1H), 3.42-3.45 (m, 1H), 3.68-3.72 (m, 1H), 3.92-3.99 (m, 1H), 5.64 (s, 1H), 6.44 (s, 2H), 9.25 (s, 1H). LC-MS [M+Na]$^+$ *m/z* 360.2 (calcd for $C_{15}H_{37}N_5O_4$, 337.18).

2.73 *Sodium (2S,5R)-2-(N-((R)-1-acetylpiperidine-2-carbonyl)carbamimidoyl)-7-oxo-1,6-diazabicyclo[3.2.1]octan-6-yl sulfate (**A22**):* Compound **A22** (170mg, 83%) as a white solid was prepared from **C22** (150 mg, 0.44 mmol) and $SO_3{\cdot}NMe_3$ (139 mg, 0.1 mmol) by using the procedure described for **A1**. ^{1}H NMR (400 MHz, D_2O): δ 1.47-1.83 (m, 6H), 1.93-2.44 (m, 7H), 3.31-3.45 (m, 1H), 3.47-3.55 (m 1H), 3.60-3.68 (m, 1H), 3.90-4.04 (m, 2H), 4.95-5.03 (m, m, 1H), 5.60 (s, 1H). LC-MS [M-Na]$^-$ *m/z* 416.1 (calcd for $C_{15}H_{22}NaN_5O_7S$, 439.11).

2.74 *tert-Butyl (2-((((2S,5R)-6-((tert-butyldimethylsilyl)oxy)-7-oxo-1,6-diazabicyclo[3.2.1]octan-2-yl)(imino)methyl)carbamoyl)thiazol-4-yl)carbamate (**B23**):* Compound **B23** (440 mg, 80%) as a white foam was prepared from 4-((tert-butoxycarbonyl)amino)thiazole-2-carboxylic acid (374 mg, 1.53 mmol) and **2** (305 mg, 1.02 mmol) by using the procedure described for **B3**. ^{1}H NMR (400 MHz, DMSO-d_6): δ0.12 (s, 6H), 0.92 (s, 9H), 1.49 (s, 9H), 1.79-1.90 (m, 2H), 2.00-2.77 (m, 2H), 3.16-3.27 (m, 1H), 3.53-3.63 (m, 1H), 4.33-4.41 (m, 1H), 6.59 (s, 2H), 7.72 (s, 1H), 11.66 (s, 1H). LC-MS [M+H]$^+$ *m/z* 525.2 (calcd for $C_{22}H_{36}N_6O_5SSi$, 524.22).

2.75 *tert-Butyl (2-((((2S,5R)-6-hydroxy-7-oxo-1,6-diazabicyclo[3.2.1]octan-2-yl)(imino)methyl)carbamoyl)thiazol-4-yl)carbamate (**C23**):* Compound **C23** (235 mg, 80%) as a white solid was prepared from **B23** (374 mg, 0.71 mmol) by using the procedure described for **C22**. ^{1}H NMR (400 MHz, DMSO-d_6): δ 1.48 (s, 9H), 1.72-1.78 (m, 1H), 1.82-1.95 (m, 2H), 2.00-2.06 (m, 1H), 3.02-3.12 (m, 1H), 3.91-3.99 (m, 1H), 4.17-4.27 (m, 1H), 5.83-6.04 (m, 1H), 6.45 (s, 2H), 7.71 (s, 1H), 9.24 (s, 1H), 11.68 (s, 1H). LC-MS [M+H]$^+$ *m/z* 411.2 (calcd for $C_{16}H_{22}N_6O_5S$, 410.14).

2.76 *Sodium (2S,5R)-2-(N-(4-aminothiazole-2-carbonyl)carbamimidoyl)-7-oxo-1,6-diazabicyclo[3.2.1]octan-6-yl sulfate (**A23**):* The starting material **C23** (315 mg, 0.77 mmol) was first treated with $SO_3{\cdot}$pyridine by using the procedure described for **A3** to get the intermediate (560 mg, 90%) as a light yellow foam (LC-MS [M-H]$^-$ *m/z* 489.1). The crude intermediate was then reacted with TFA (2.1 mL) in anhydrous CH_2Cl_2 (7 mL) at 0 °C and stirred for 3.5 hours at 0 °C. Another portion of TFA (1 mL) was added to the reaction mixture, and stirring continued at 0 °C for additional 6.5 hours. The reaction mixture was concentrated under reduced pressure to give a residue. The residue was dissolved with CH_2Cl_2 (20 mL) and extracted with water (2 × 10 mL). The aqueous layer was freeze-dried to give a solid residue, which was purified by Dowex-50wx Na^+ resin, using water as an elution solvent to give **A23** (100 mg, 35%) as a white powder. ^{1}H NMR (400 MHz, D_2O): δ 1.90-2.01 (m, 2H), 2.06-2.16 (m, 2H), 3.31-3.45 (m, 1H), 3.93-4.16 (m, 1H), 4.18-4.41 (m, 1H), 5.58 (d, J = 5.9 Hz, 1H), 7.10 (s, 1H). LC-MS [M-Na]$^-$ *m/z* 389.0 (calcd for $C_{11}H_{13}NaN_6O_6S_2$, 412.02).

3 Determination of antibacterial activity

A standard procedure was employed for these studies using the broth microdilution method according to the guidelines of the Clinical Laboratories and Standards Institute[1]. In a typical experiment, meropenem as a test antibiotic compound was dissolved in DMSO and diluted in microbial growth medium (Mueller-Hinton Broth II, cation adjusted) to a final concentration range of 0.125 mg/L to 64 mg/L in serial two-fold dilution. In all cases the final DMSO concentration was less than 0.5%. Bacteria were added to 96-well microtiter plates containing the serial two-fold dilutions of the compounds; the final cell density was approximately 5×10^5 colony forming units/mL (CFU/mL). The plates were incubated at 37 °C for 18–24 hours and read visually. The MIC of the test compound that inhibited visible growth of the bacteria was recorded. The same assay conditions were used when the compounds **A1**–**23** and avibactam alone and as a combination with test meropenem as an antibiotic compound (as two controls) were tested for MIC (mg/L). While meropenem was serially diluted as described above, a constant concentration (4 mg/L) of the inhibitor compounds **A1**–**23** and avibactam as reference inhibitor were used. The test results are listed in Table 1.

4 NMR Spectra of final compounds A1–23

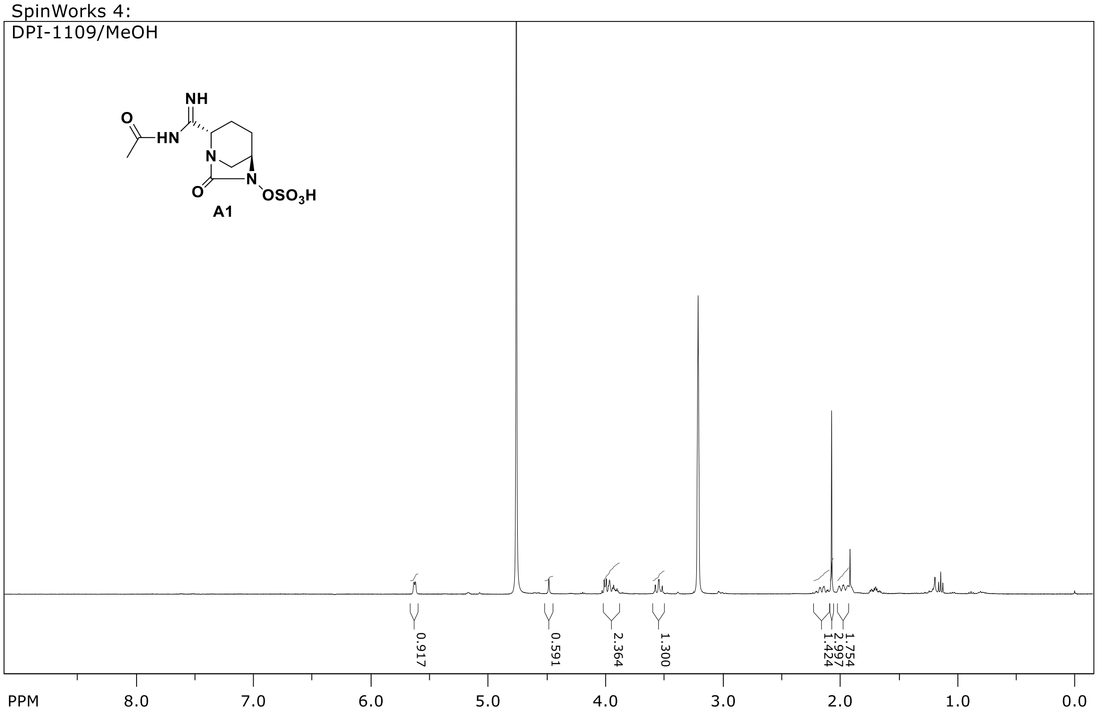

file: ...PI-1109\01YG1020-125-016-01\10\fid expt: <zg30>
transmitter freq.: 400.132471 MHz
time domain size: 65536 points
width: 8196.72 Hz = 20.4850 ppm = 0.125072 Hz/pt
number of scans: 16

freq. of 0 ppm: 400.130047 MHz
processed size: 65536 complex points
LB: 0.300 GF: 0.0000

SpinWorks 4:

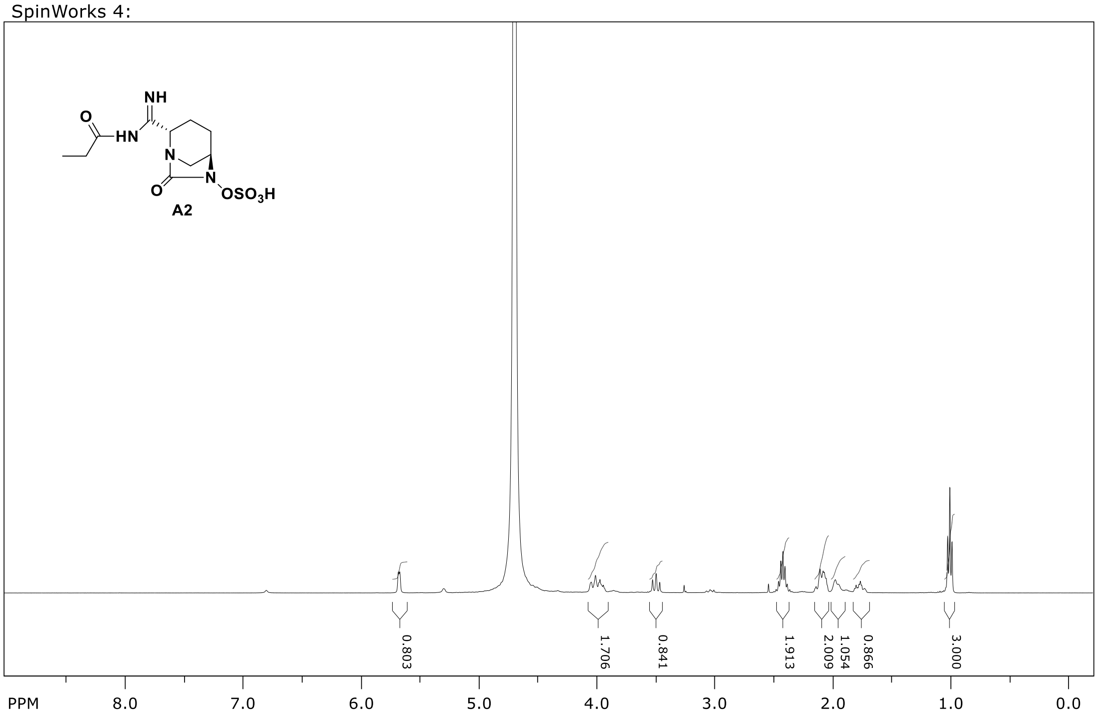

file: ...ort\Haikang\01HY1035-013-01\10\fid expt: <zg30>
transmitter freq.: 400.132471 MHz
time domain size: 65536 points
width: 8196.72 Hz = 20.4850 ppm = 0.125072 Hz/pt
number of scans: 32

freq. of 0 ppm: 400.130000 MHz
processed size: 65536 complex points
LB: 0.300 GF: 0.0000

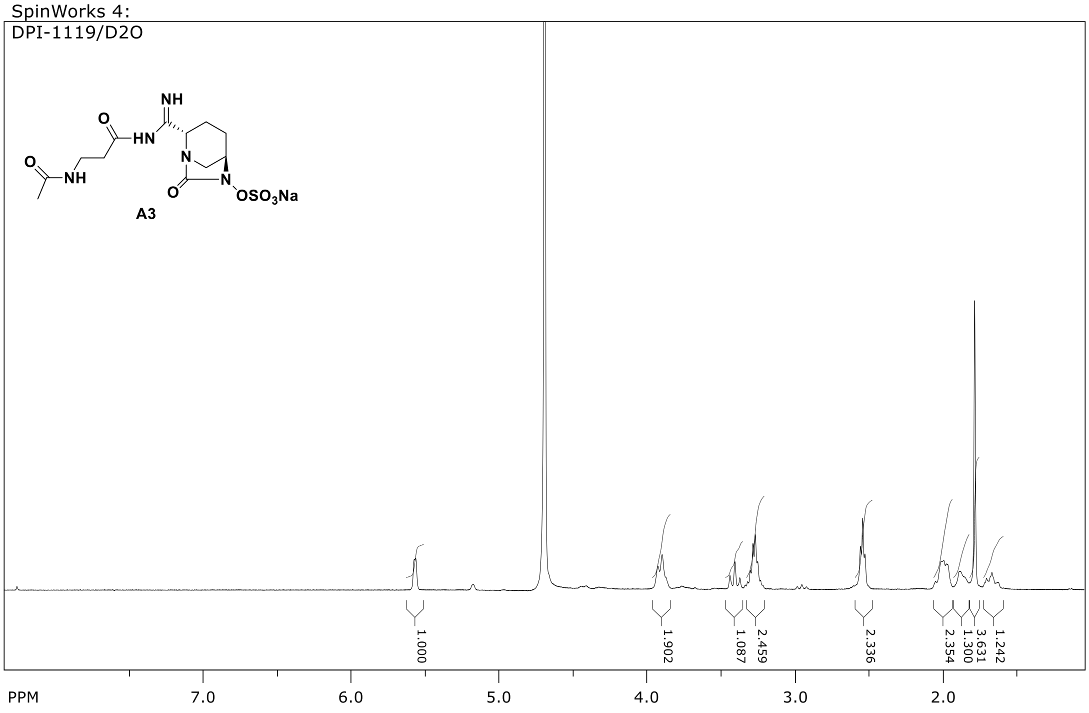

file: ...-2\DPI-1119\01HY1023-199-01\10\fid expt: <zg30>
transmitter freq.: 400.132471 MHz
time domain size: 65536 points
width: 8196.72 Hz = 20.4850 ppm = 0.125072 Hz/pt
number of scans: 32

freq. of 0 ppm: 400.130000 MHz
processed size: 65536 complex points
LB: 0.300 GF: 0.0000

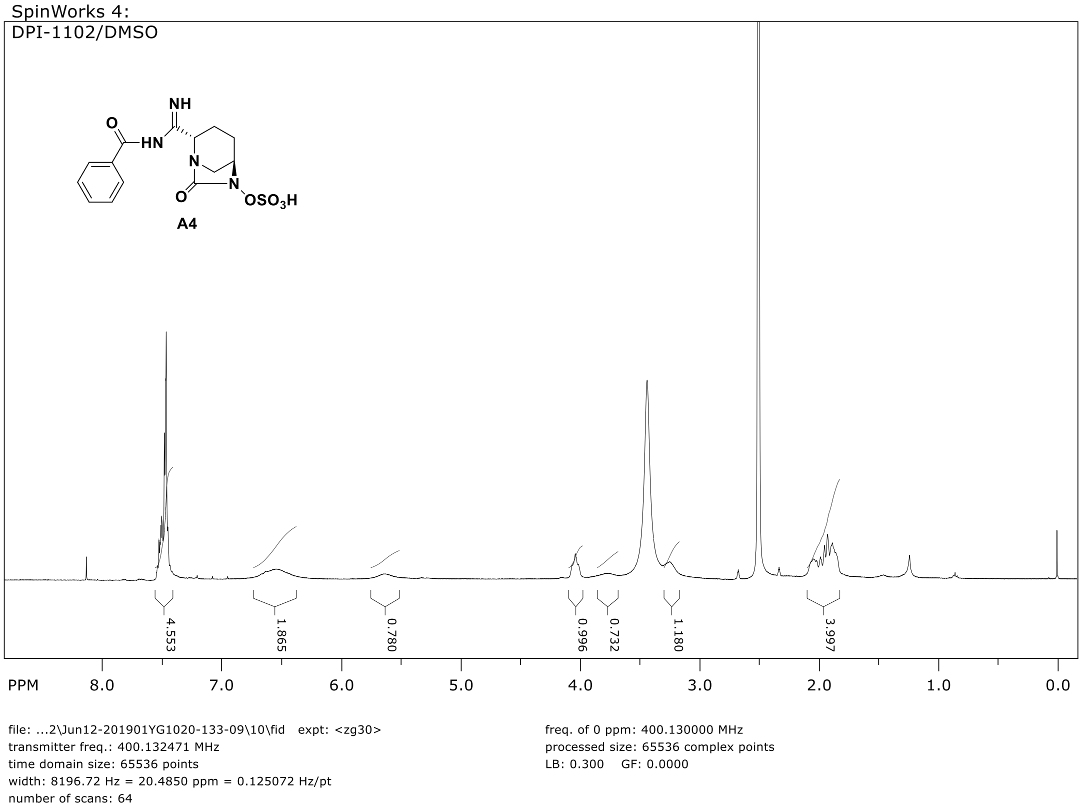
SpinWorks 4:
DPI-1102/DMSO
NH
O
HN
N
N
O
OSO3H
A4
4.553
1.865
0.780
0.996
0.732
1.180
3.997
PPM
8.0
7.0
6.0
5.0
4.0
3.0
2.0
1.0
0.0
file: ...2\Jun12-201901YG1020-133-09\10\fid expt: <zg30>
transmitter freq.: 400.132471 MHz
time domain size: 65536 points
width: 8196.72 Hz = 20.4850 ppm = 0.125072 Hz/pt
number of scans: 64
freq. of 0 ppm: 400.130000 MHz
processed size: 65536 complex points
LB: 0.300 GF: 0.0000

SpinWorks 4:

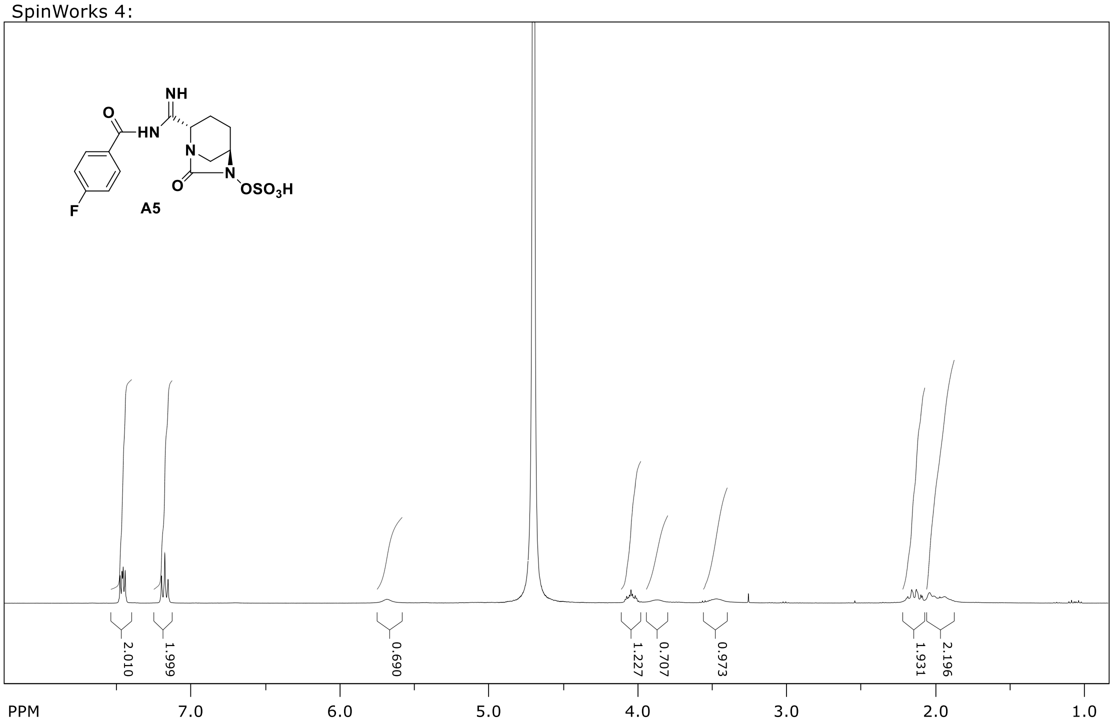

file: ...ort\Haikang\01HY1035-006-01\10\fid   expt: <zg30>
transmitter freq.: 400.132471 MHz
time domain size: 65536 points
width: 8196.72 Hz = 20.4850 ppm = 0.125072 Hz/pt
number of scans: 32

freq. of 0 ppm: 400.130000 MHz
processed size: 65536 complex points
LB: 0.300   GF: 0.0000

SpinWorks 4:

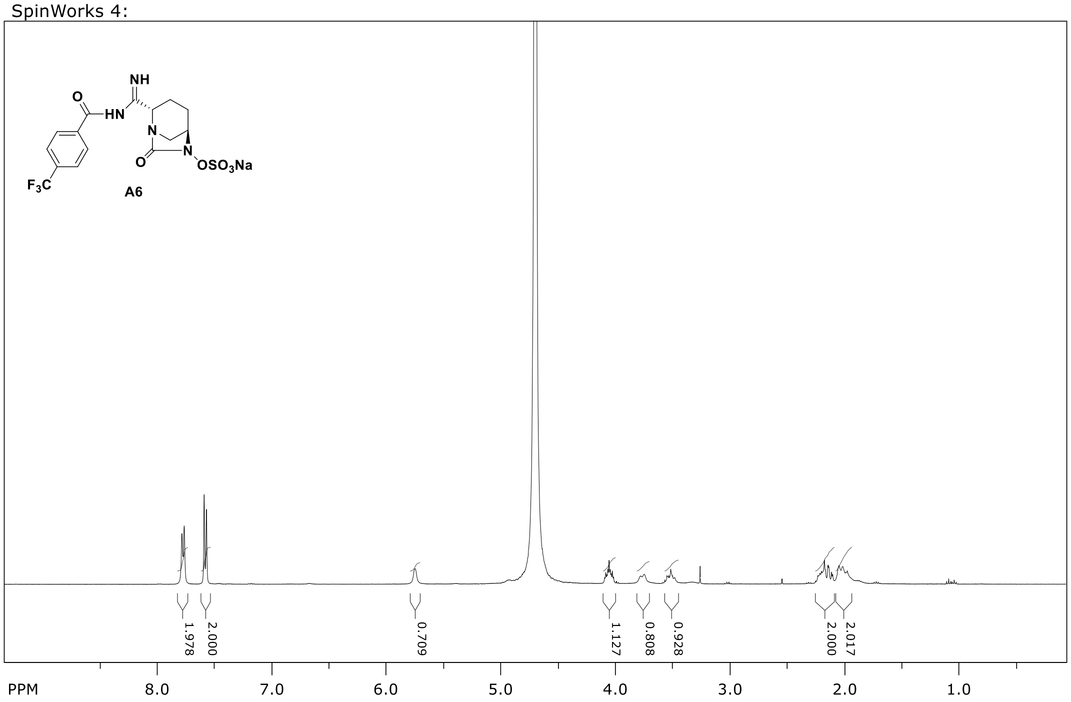

file: ...ort\Haikang\01HY1035-007-01\10\fid expt: <zg30>
transmitter freq.: 400.132471 MHz
time domain size: 65536 points
width: 8196.72 Hz = 20.4850 ppm = 0.125072 Hz/pt
number of scans: 32

freq. of 0 ppm: 400.130000 MHz
processed size: 65536 complex points
LB: 0.300 GF: 0.0000

SpinWorks 4:

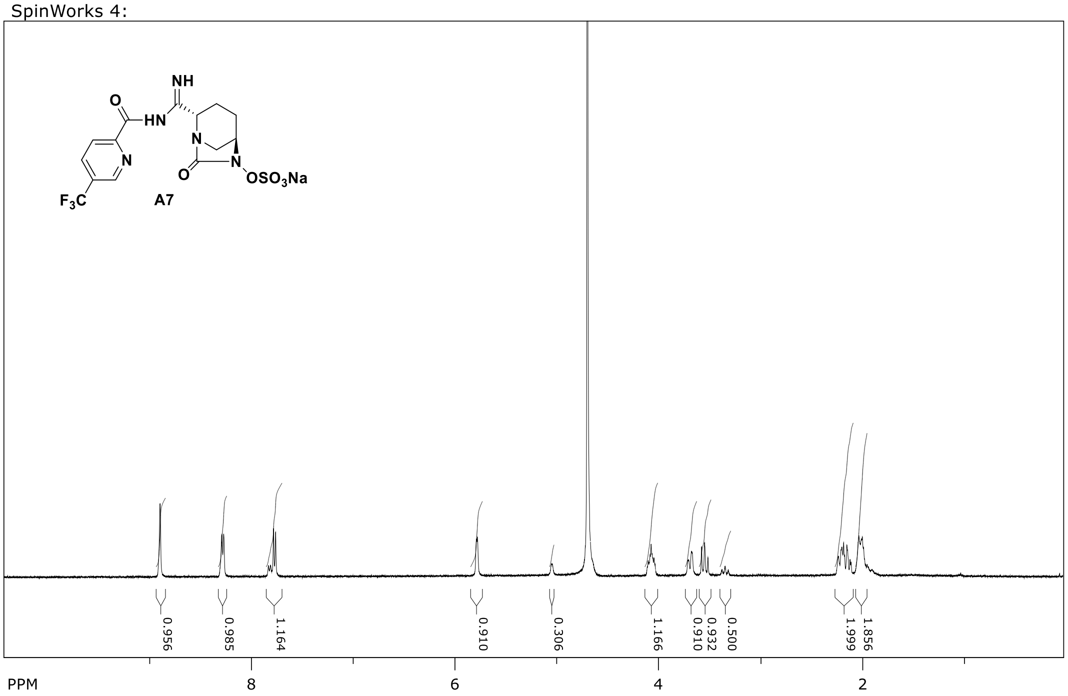

file: ...Zhai Lijuan\01LZ1032-044-01\10\fid expt: <zg30>
transmitter freq.: 400.132471 MHz
time domain size: 65536 points
width: 8196.72 Hz = 20.4850 ppm = 0.125072 Hz/pt
number of scans: 32

freq. of 0 ppm: 400.130000 MHz
processed size: 65536 complex points
LB: 0.300 GF: 0.0000

SpinWorks 4:

DPI-1106/DMSO

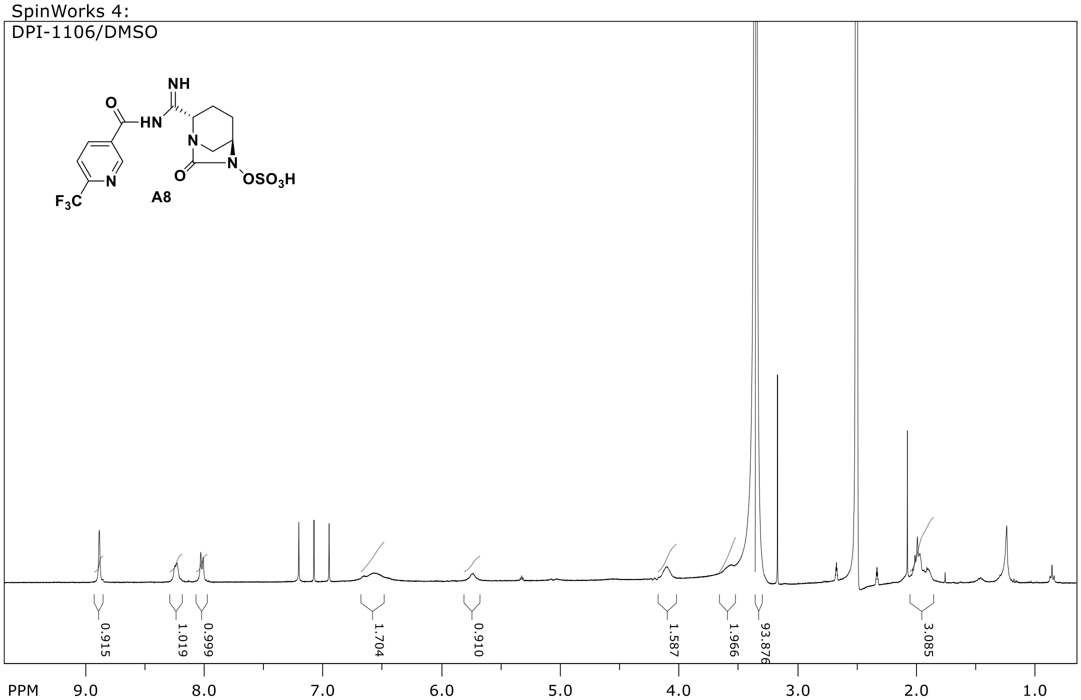

file: ...6\Aug05-201901YG1020-149-09\10\fid expt: <zg30>
transmitter freq.: 400.132471 MHz
time domain size: 65536 points
width: 8196.72 Hz = 20.4850 ppm = 0.125072 Hz/pt
number of scans: 64

freq. of 0 ppm: 400.130002 MHz
processed size: 65536 complex points
LB: 0.300 GF: 0.0000

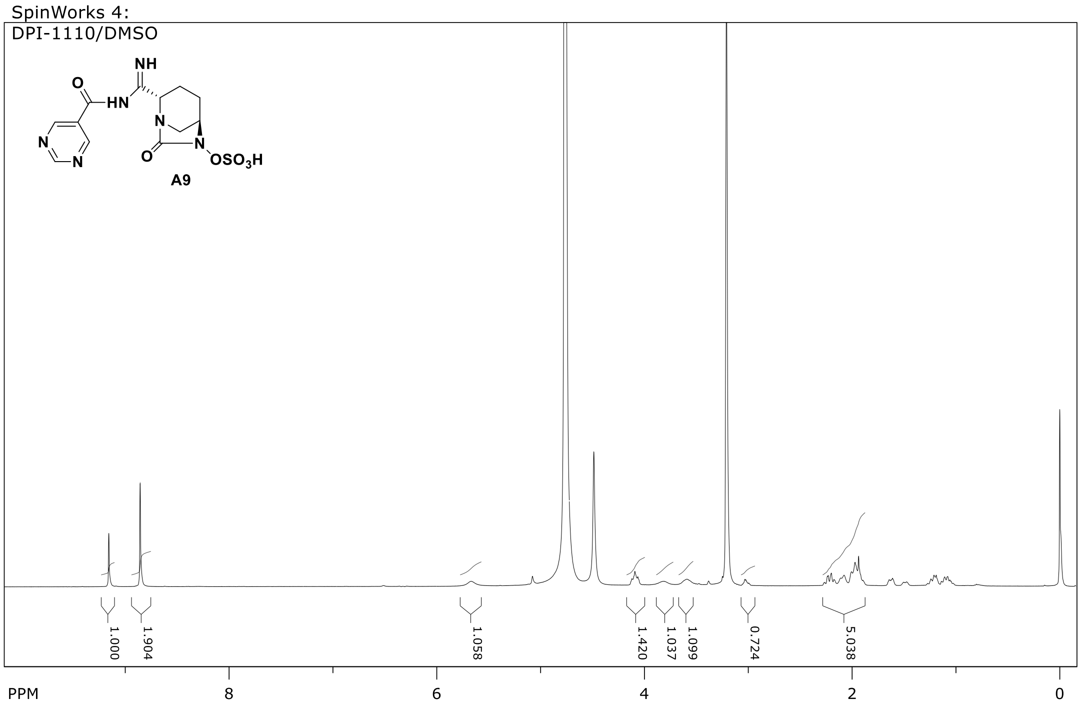

file: ...bitor-2\DPI-1110\Sep20-2019\10\fid expt: <zg30>
transmitter freq.: 400.132471 MHz
time domain size: 65536 points
width: 8196.72 Hz = 20.4850 ppm = 0.125072 Hz/pt
number of scans: 1024

freq. of 0 ppm: 400.130047 MHz
processed size: 65536 complex points
LB: 0.300 GF: 0.0000

SpinWorks 4:

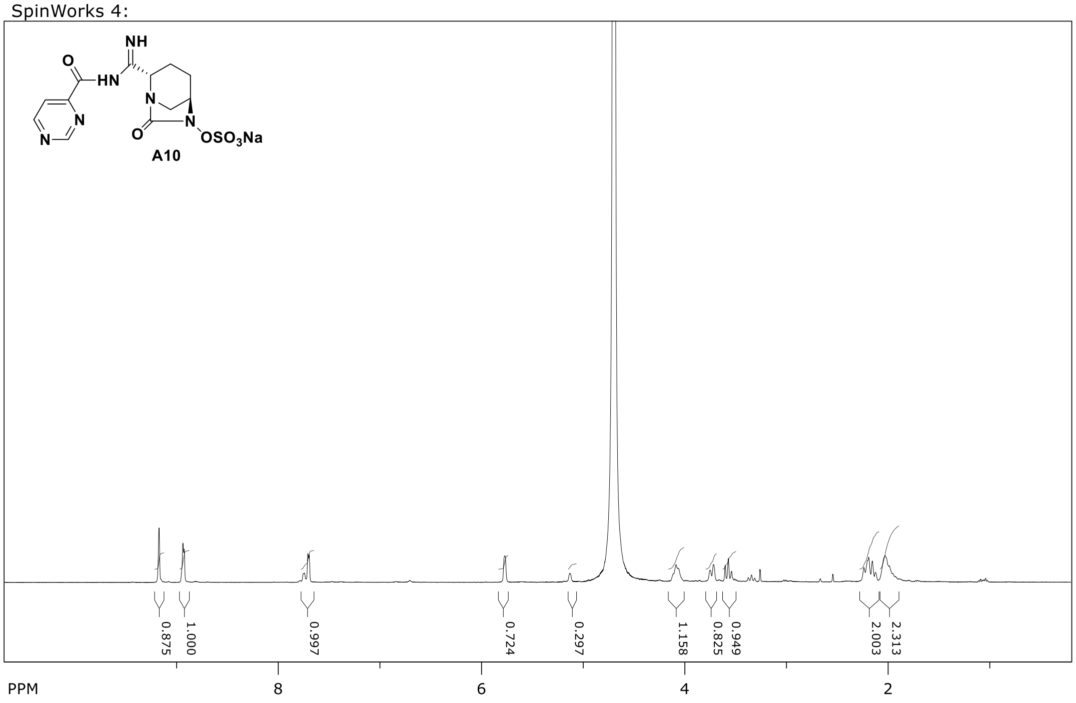

file: ...ort\Haikang\01HY1035-010-01\10\fid expt: <zg30>
transmitter freq.: 400.132471 MHz
time domain size: 65536 points
width: 8196.72 Hz = 20.4850 ppm = 0.125072 Hz/pt
number of scans: 32

freq. of 0 ppm: 400.130000 MHz
processed size: 65536 complex points
LB: 0.300 GF: 0.0000

SpinWorks 4:

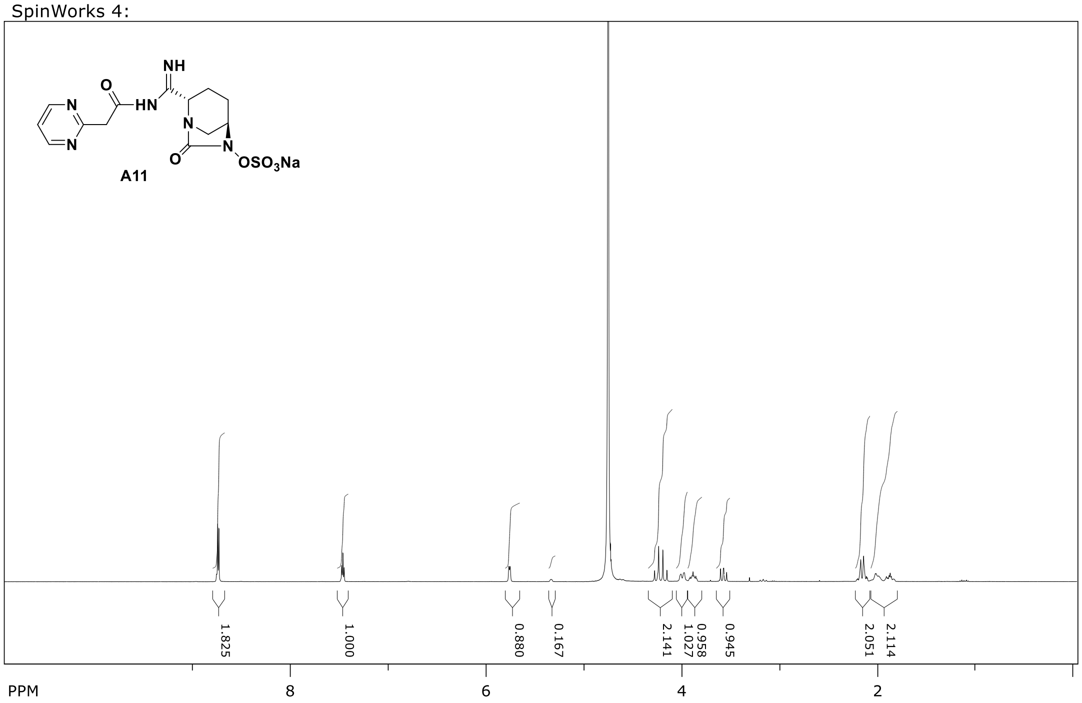

file: ...t_Gao_HK_ZL\01HY1023-257-01\10\fid expt: <zg30>
transmitter freq.: 400.132471 MHz
time domain size: 65536 points
width: 8196.72 Hz = 20.4850 ppm = 0.125072 Hz/pt
number of scans: 32

freq. of 0 ppm: 400.129980 MHz
processed size: 65536 complex points
LB: 0.300 GF: 0.0000

SpinWorks 4: DPI-1113/D2O

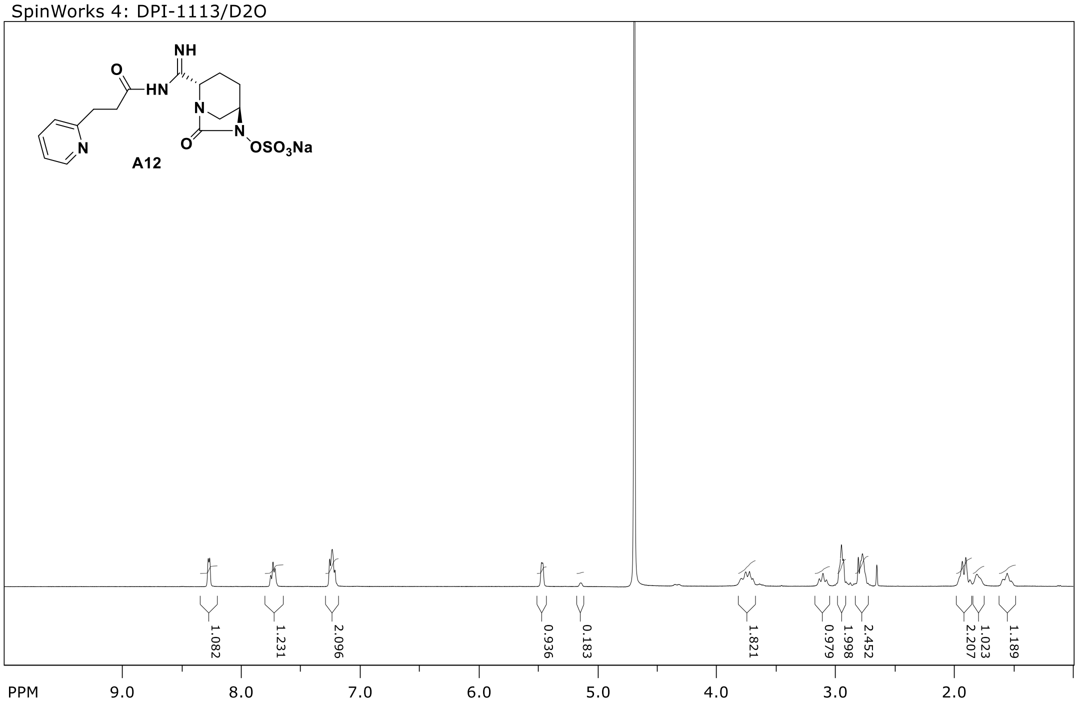

file: ...-2\DPI-1113\01DT1029-089-01\10\fid expt: <zg30>
transmitter freq.: 400.132471 MHz
time domain size: 65536 points
width: 8196.72 Hz = 20.4850 ppm = 0.125072 Hz/pt
number of scans: 32

freq. of 0 ppm: 400.130000 MHz
processed size: 65536 complex points
LB: 0.300 GF: 0.0000

SpinWorks 4:

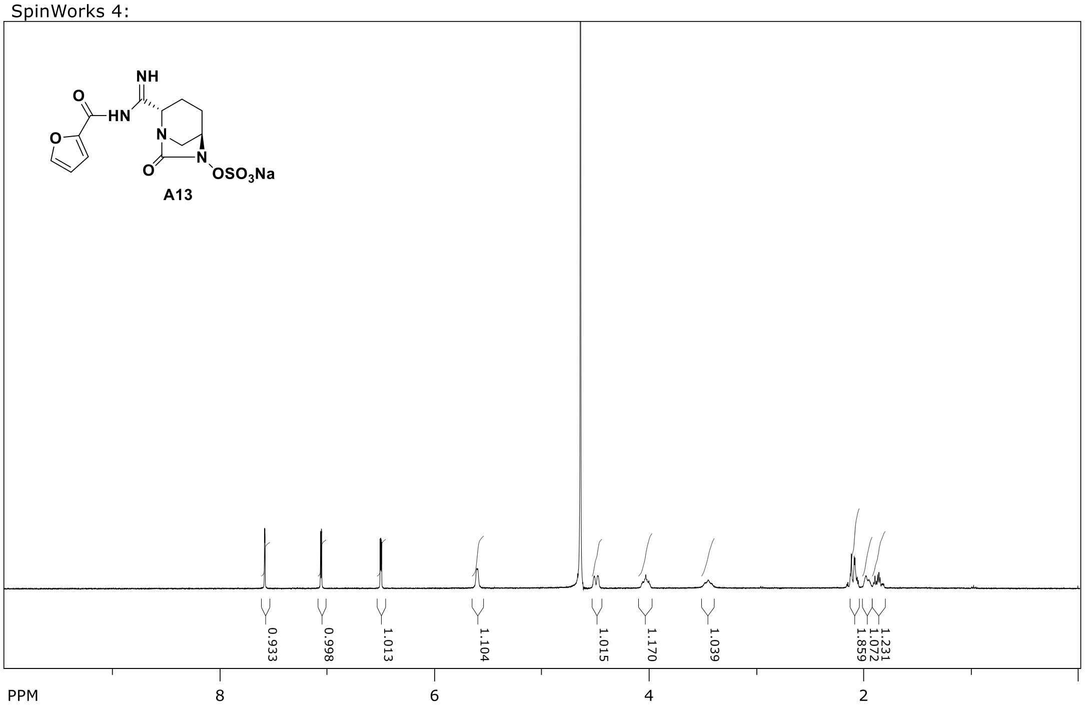

file: ...Zhai Lijuan\01LZ1032-042-01\40\fid expt: <zg30>
transmitter freq.: 400.132471 MHz
time domain size: 65536 points
width: 8196.72 Hz = 20.4850 ppm = 0.125072 Hz/pt
number of scans: 16

freq. of 0 ppm: 400.130024 MHz
processed size: 65536 complex points
LB: 0.300 GF: 0.0000

SpinWorks 4: DPI-1121/D2O

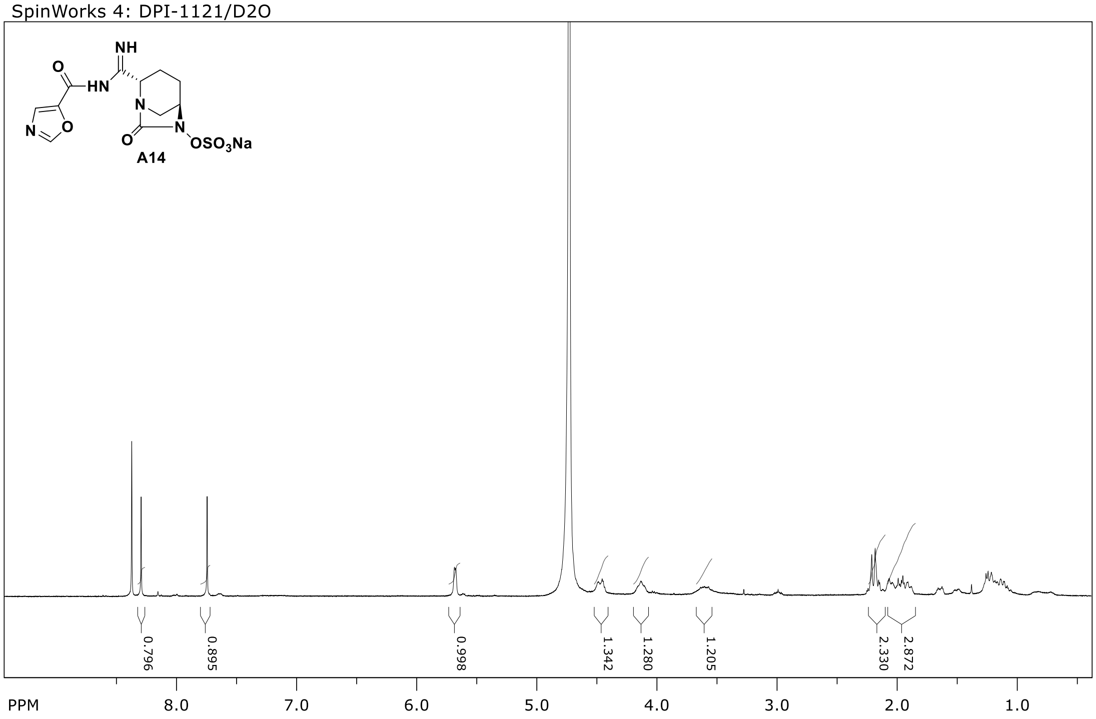

file: ...-2\DPI-1121\01JJ1026-020-01\10\fid expt: <zg30>
transmitter freq.: 400.132471 MHz
time domain size: 65536 points
width: 8196.72 Hz = 20.4850 ppm = 0.125072 Hz/pt
number of scans: 32

freq. of 0 ppm: 400.129987 MHz
processed size: 65536 complex points
LB: 0.300 GF: 0.0000

SpinWorks 4: DPI-1120/D2O

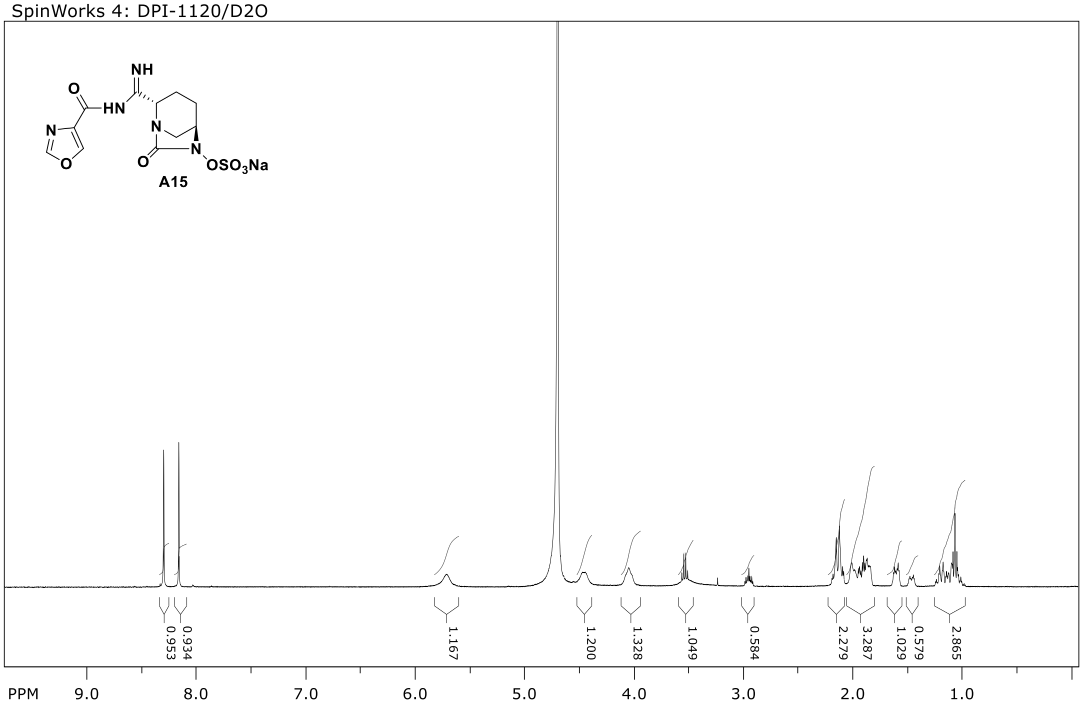

file: ...-2\DPI-1120\01JJ1026-023-01\10\fid expt: <zg30>
transmitter freq.: 400.132471 MHz
time domain size: 65536 points
width: 8196.72 Hz = 20.4850 ppm = 0.125072 Hz/pt
number of scans: 32

freq. of 0 ppm: 400.130000 MHz
processed size: 65536 complex points
LB: 0.300 GF: 0.0000

SpinWorks 4:

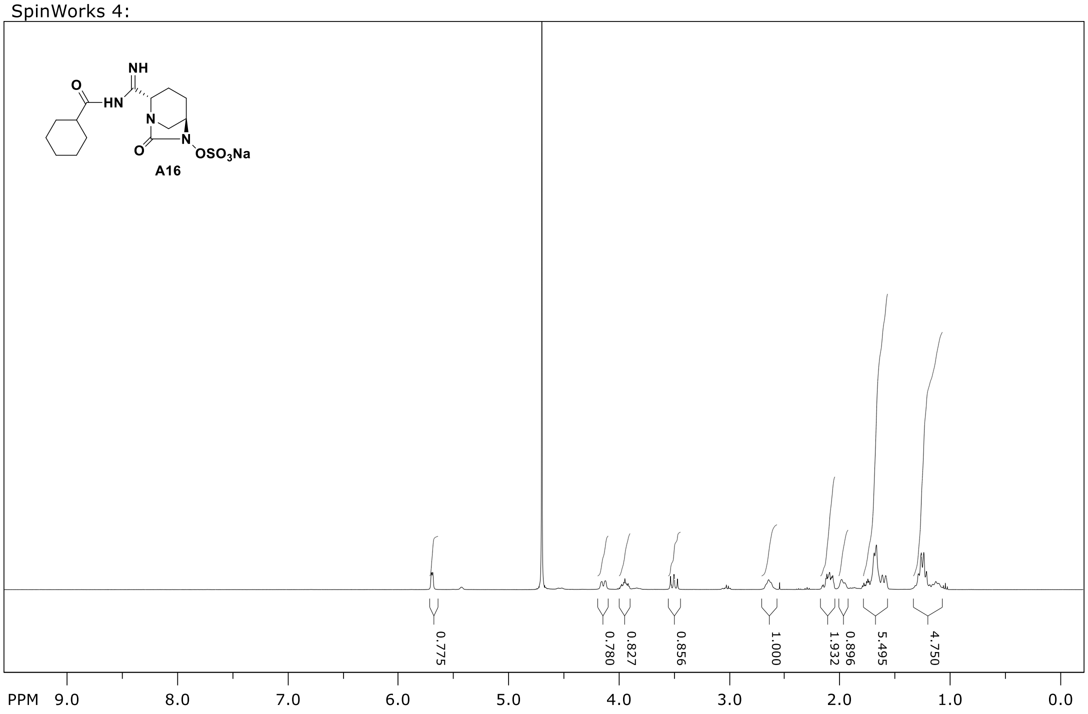

file: ...ort\Haikang\01HY1035-016-01\10\fid expt: <zg30>
transmitter freq.: 400.132471 MHz
time domain size: 65536 points
width: 8196.72 Hz = 20.4850 ppm = 0.125072 Hz/pt
number of scans: 32

freq. of 0 ppm: 400.130000 MHz
processed size: 65536 complex points
LB: 0.300 GF: 0.0000

SpinWorks 4:

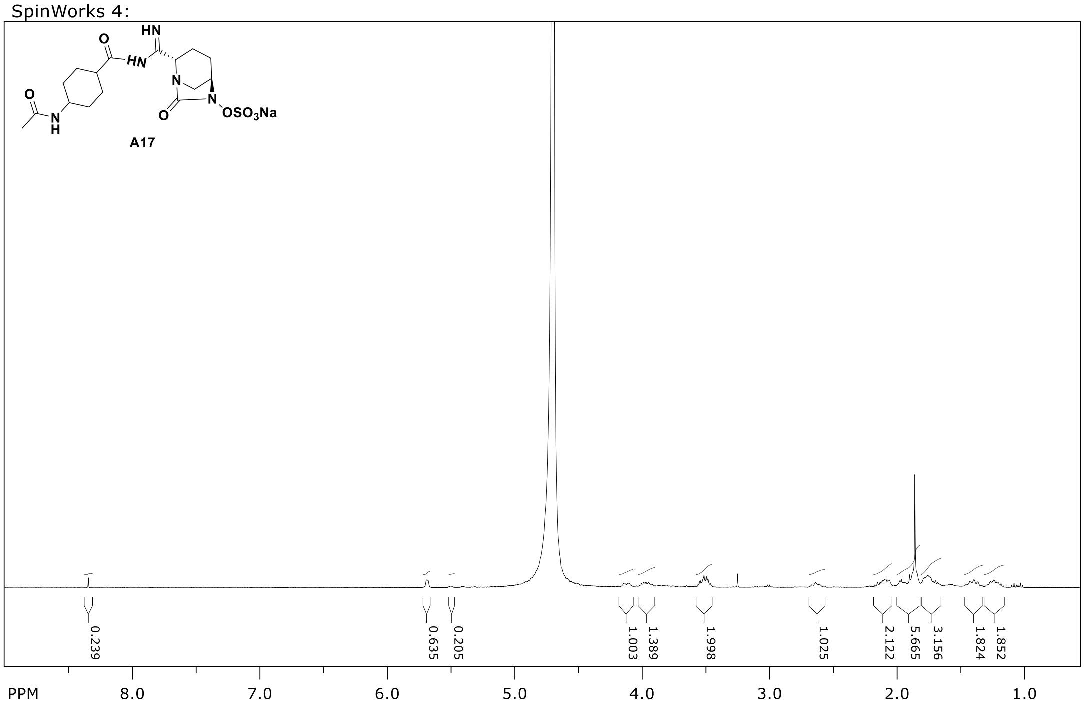

file: ...lder20200623\01YG1031-31-01\10\fid expt: <zg30>
transmitter freq.: 400.132471 MHz
time domain size: 65536 points
width: 8196.72 Hz = 20.4850 ppm = 0.125072 Hz/pt
number of scans: 32

freq. of 0 ppm: 400.130000 MHz
processed size: 65536 complex points
LB: 0.300 GF: 0.0000

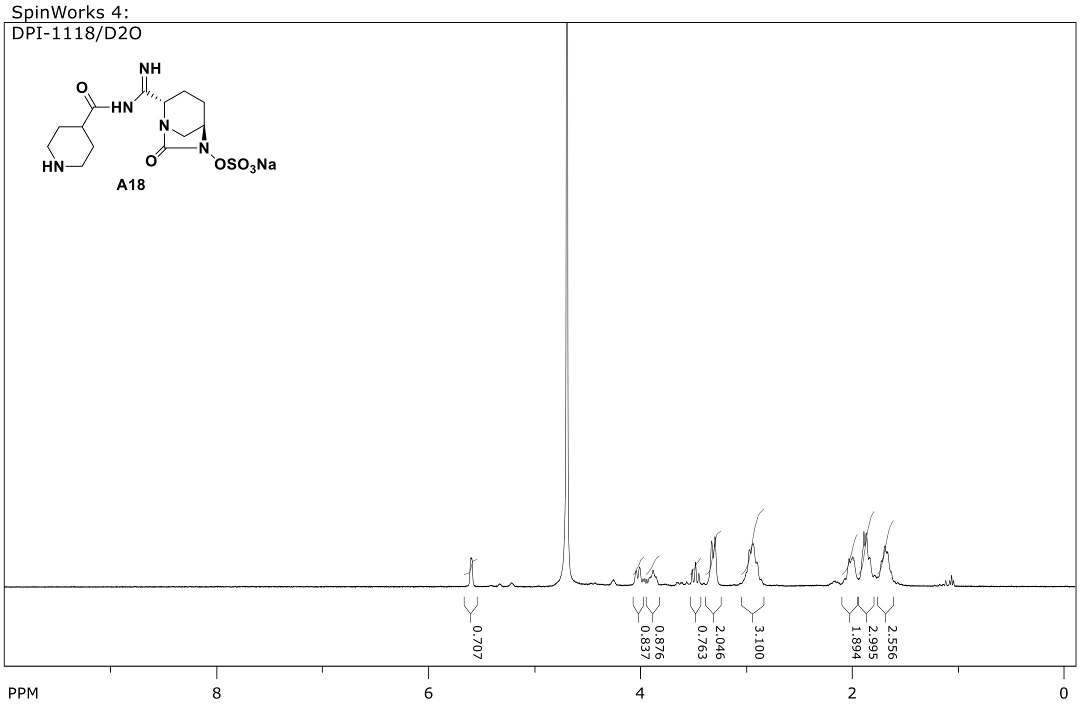

file: ...-2\DPI-1118\01HY1023-204-01\10\fid expt: <zg30>
transmitter freq.: 400.132471 MHz
time domain size: 65536 points
width: 8196.72 Hz = 20.4850 ppm = 0.125072 Hz/pt
number of scans: 16

freq. of 0 ppm: 400.130000 MHz
processed size: 65536 complex points
LB: 0.300 GF: 0.0000

SpinWorks 4:

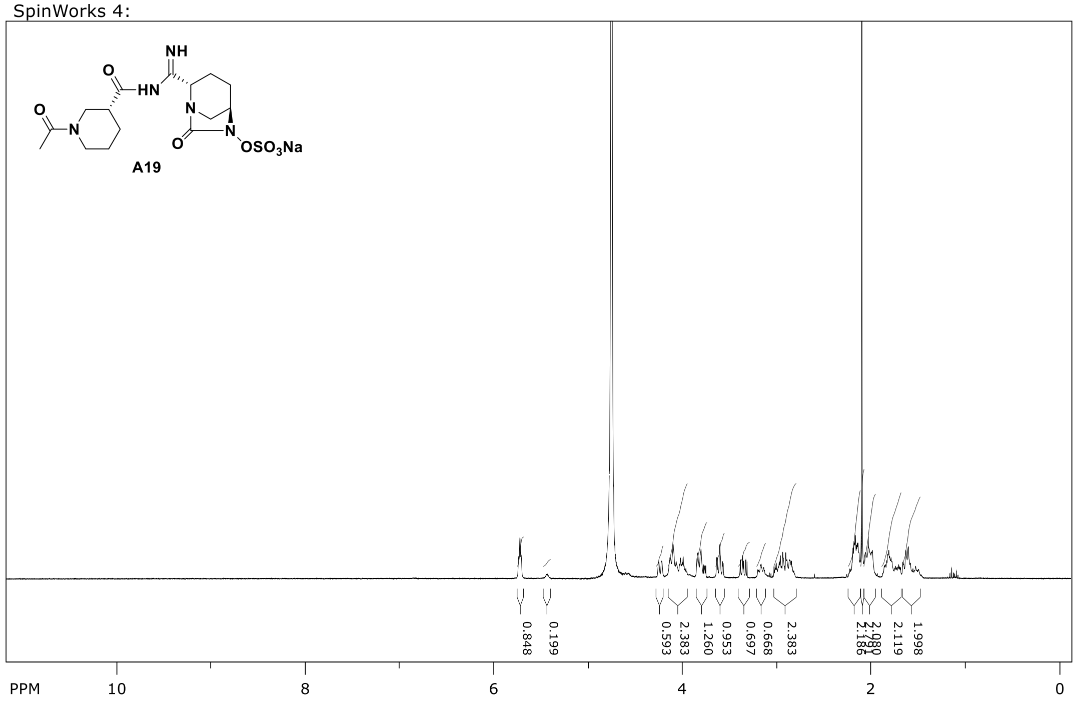

file: ...t_Gao_HK_ZL\01HY1023-238-01\10\fid expt: <zg30>
transmitter freq.: 400.132471 MHz
time domain size: 65536 points
width: 8196.72 Hz = 20.4850 ppm = 0.125072 Hz/pt
number of scans: 32

freq. of 0 ppm: 400.129980 MHz
processed size: 65536 complex points
LB: 0.300 GF: 0.0000

SpinWorks 4:

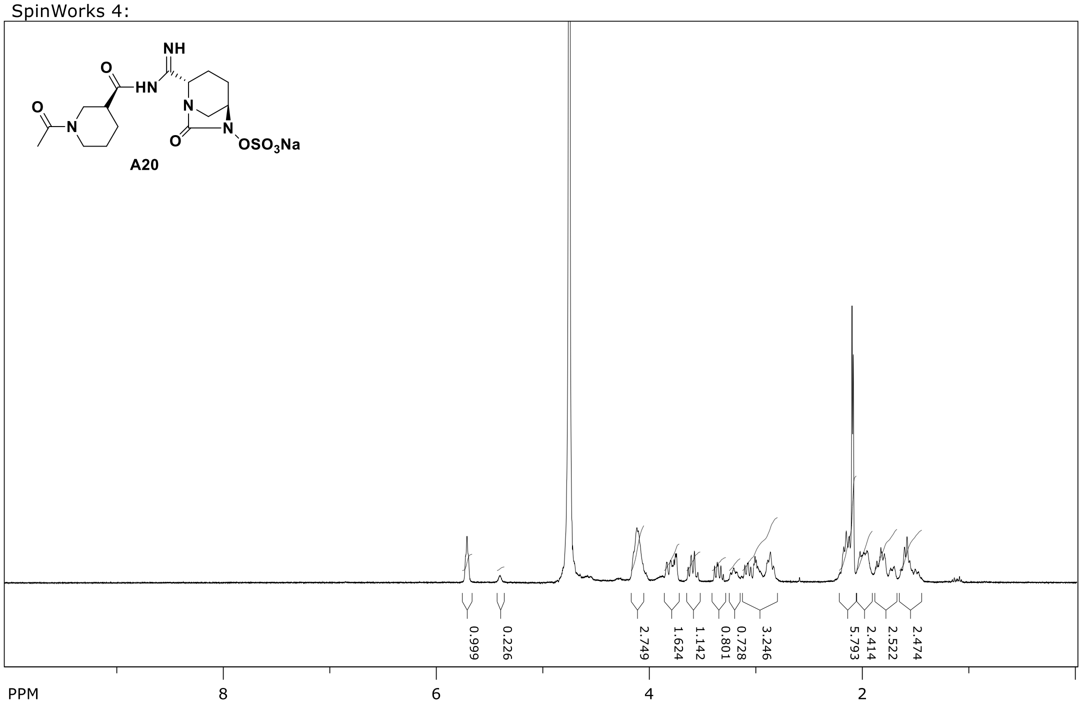

file: ...t_Gao_HK_ZL\01HY1023-236-01\10\fid expt: <zg30>
transmitter freq.: 400.132471 MHz
time domain size: 65536 points
width: 8196.72 Hz = 20.4850 ppm = 0.125072 Hz/pt
number of scans: 16

freq. of 0 ppm: 400.129979 MHz
processed size: 65536 complex points
LB: 0.300 GF: 0.0000

SpinWorks 4:

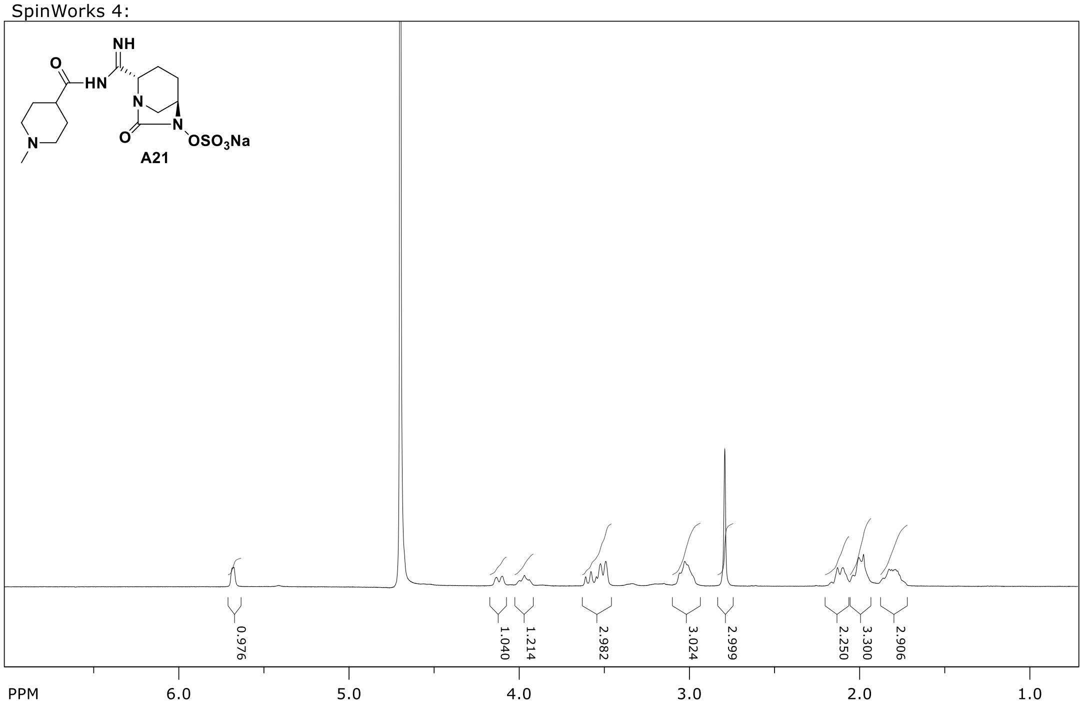

file: ...ort\Haikang\01JJ1011-037-01\10\fid expt: <zg30>
transmitter freq.: 400.132471 MHz
time domain size: 65536 points
width: 8196.72 Hz = 20.4850 ppm = 0.125072 Hz/pt
number of scans: 128

freq. of 0 ppm: 400.130000 MHz
processed size: 65536 complex points
LB: 0.300 GF: 0.0000

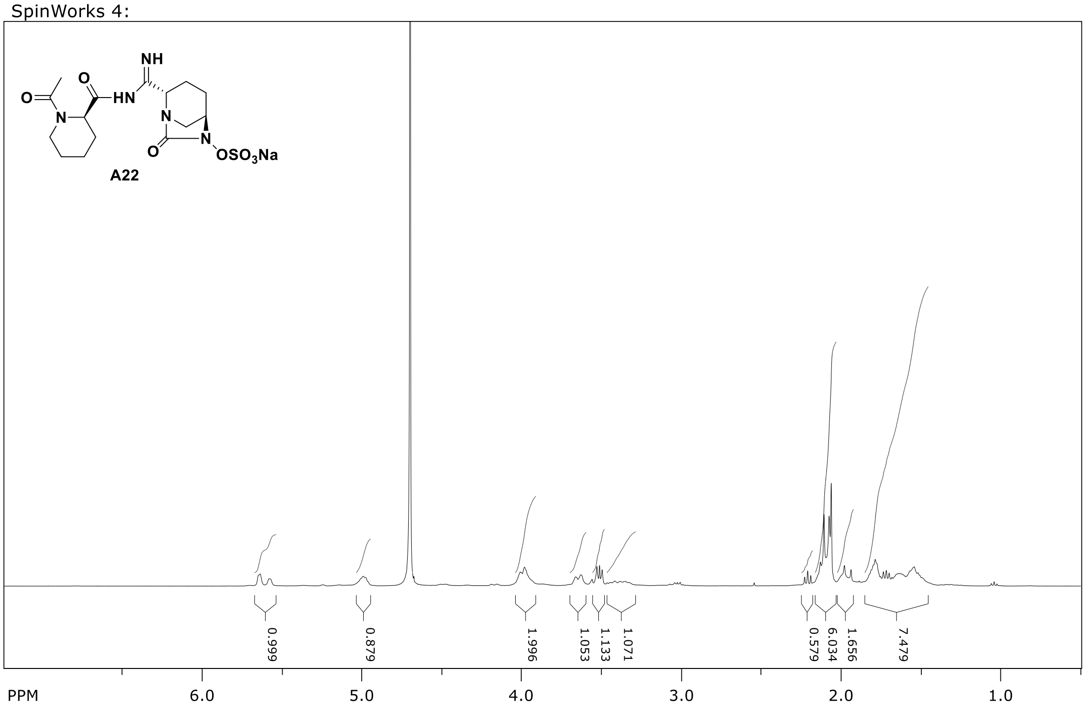

file: ...ort\Haikang\01HY1035-014-01\10\fid expt: <zg30>
transmitter freq.: 400.132471 MHz
time domain size: 65536 points
width: 8196.72 Hz = 20.4850 ppm = 0.125072 Hz/pt
number of scans: 32

freq. of 0 ppm: 400.130000 MHz
processed size: 65536 complex points
LB: 0.300 GF: 0.0000

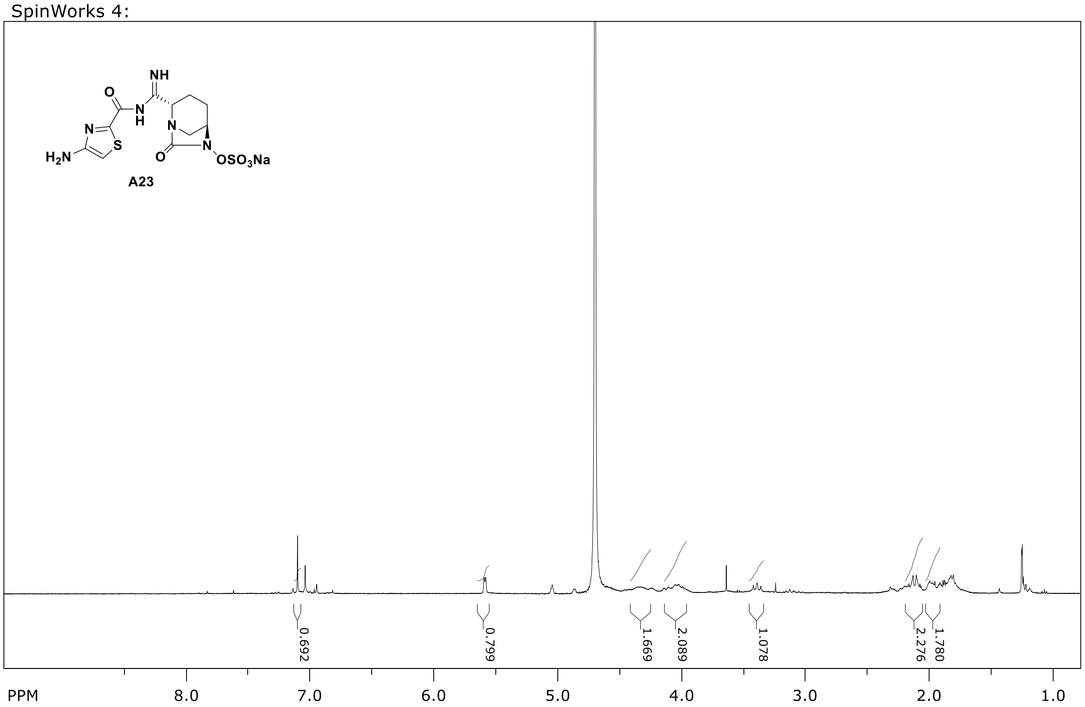

file: ...der20200602\01HY1023-227-01\10\fid expt: <zg30>
transmitter freq.: 400.132471 MHz
time domain size: 65536 points
width: 8196.72 Hz = 20.4850 ppm = 0.125072 Hz/pt
number of scans: 32

freq. of 0 ppm: 400.130000 MHz
processed size: 65536 complex points
LB: 0.300 GF: 0.0000

References

1. M. A. Wikler, *Methods for Dilution Antimicrobial Susceptibility Tests for Bacteria that Grow Aerobically; Approved Standard*, Clinical and Laboratory Standards Institute Wayne, Pa., USA, 2009.
